# Application of Benchtop NMR for Metabolomics Study Using Feces of Mice with DSS-Induced Colitis

**DOI:** 10.3390/metabo13050611

**Published:** 2023-04-28

**Authors:** Zihao Song, Yuki Ohnishi, Seiji Osada, Li Gan, Jiaxi Jiang, Zhiyan Hu, Hiroyuki Kumeta, Yasuhiro Kumaki, Yuki Yokoi, Kiminori Nakamura, Tokiyoshi Ayabe, Kazuo Yamauchi, Tomoyasu Aizawa

**Affiliations:** 1 Laboratory of Protein Science, Graduate School of Life Science, Hokkaido University, Sapporo 060-0808, Japan; 2 Nakayama Co., Ltd., Tsukuba 300-2651, Japan; 3 Advanced NMR Facility, Faculty of Advanced Life Science, Hokkaido University, Sapporo 060-0808, Japan; 4 High-Resolution NMR Laboratory, Graduate School of Science, Hokkaido University, Sapporo 060-0810, Japan; 5 Innate Immunity Laboratory, Graduate School of Life Science, Hokkaido University, Sapporo 060-0808, Japan; 6 Instrumental Analysis Section, Okinawa Institute of Science and Technology, Onna 904-0495, Japan

**Keywords:** benchtop NMR, metabolomics, DSS-induced mice, feces

## Abstract

Nuclear magnetic resonance (NMR)-based metabolomics, which comprehensively measures metabolites in biological systems and investigates their response to various perturbations, is widely used in research to identify biomarkers and investigate the pathogenesis of underlying diseases. However, further applications of high-field superconducting NMR for medical purposes and field research are restricted by its high cost and low accessibility. In this study, we applied a low-field, benchtop NMR spectrometer (60 MHz) employing a permanent magnet to characterize the alterations in the metabolic profile of fecal extracts obtained from dextran sodium sulfate (DSS)-induced ulcerative colitis model mice and compared them with the data acquired from high-field NMR (800 MHz). Nineteen metabolites were assigned to the 60 MHz ^1^H NMR spectra. Non-targeted multivariate analysis successfully discriminated the DSS-induced group from the healthy control group and showed high comparability with high-field NMR. In addition, the concentration of acetate, identified as a metabolite with characteristic behavior, could be accurately quantified using a generalized Lorentzian curve fitting method based on the 60 MHz NMR spectra.

## 1. Introduction

Metabolomics targets the comprehensive measurement of large numbers of metabolites that are the downstream products of genes, transcripts, and protein functions. It can provide insight into the biological phenotype by identifying the fluctuations of metabolites in response to drugs, the environment, and genetic modulations [1]. Thus, metabolomics studies have been widely applied to identify key biomarkers and investigate the pathogenesis of various human diseases [2], including cardiovascular, liver, respiratory, neurological, gut diseases and cancer [3,4,5,6,7,8].

Along with mass spectroscopy, high-field NMR spectrometry based on superconducting magnets has been one of the most routinely used techniques for metabolomics studies, owing to its inherent advantages of being non-destructive, requiring a short analysis time and less sample preparation [9]. Generally, 600 MHz NMR spectrometers are considered the “recommended” instrument, which balances the field strength, resolution, and cost [10,11]. In addition, magnets with higher field strengths, such as 700 MHz and 800 MHz instruments, have been used to achieve better sensitivity and signal resolution. However, the applications of NMR-based metabolomics for medical purposes, including diagnosis, prognosis, and tracing of the recovery process, are restricted and far from routine utilization because of their low accessibility. In particular, superconducting magnets with higher field strengths result in the constantly increasing size and cost of NMR spectrometers. In addition to the substantial investment in equipment, specific facilities, cryogenic fluid maintenance, and well-trained technical staff are essential, thus limiting their accessibility in field applications [12].

The recently developed cryogen-free, low-field benchtop NMR spectrometer employing compact permanent magnets may solve these problems and represent a new approach for metabolomics studies, benefiting from its small size and low running cost. In the past, the permanent magnets were produced in small sizes at the expense of field homogeneity and could only be used to measure relaxation times and diffusion coefficients, known as NMR relaxometry or time-domain NMR. With the miniaturization of permanent magnets and advancements in modern electronics, low-field benchtop NMR technology has achieved significantly improved sensitivity and spectral quality [13]. Furthermore, it has enabled the adoption of common NMR methodologies such as solvent suppression, which is an essential issue for NMR measurement of biological samples that contain water-based solvents [14,15].

Previous studies have reported the applicability of benchtop NMR in food science, organic chemistry, and material science [16,17,18,19]. In metabolomics, the metabolic signature of type 2 diabetes has been profiled using urine samples with high reproducibility [20,21,22]. Moreover, tuberculosis in both humans and bovines was detected and differentiated by benchtop NMR-acquired metabolomic fingerprinting using urine and plasma [23,24]. Nevertheless, it should be noted that the feasibility of benchtop NMR for metabolomics studies has not been universally verified, as the existing reports are few, and the shortcomings of low sensitivity and low resolution need to be solved.

In this study, we investigated the potential application of benchtop NMR for fecal metabolomics, which has been increasingly studied using superconducting high-field NMR, as the profiling of fecal metabolome provides a functional readout of gut microbial activity and a variety of diseases, such as inflammatory bowel diseases (IBD) [25].

IBD, mainly comprised of Crohn’s disease and ulcerative colitis, is characterized by chronic recurring inflammation in the gastrointestinal tract [26]. It has been estimated that more than 6.8 million individuals were affected by IBD globally by 2017, and the number of prevalent cases is rising [27]. To elucidate the underlying pathogenesis of human IBD and identify potential therapeutic targets, rodent models have been developed, such as the chemically induced dextran sodium sulfate (DSS) colitis model and genetically modified Interleukin 10 knock-out (IL-10 ^−/−^) mice [28,29]. Although the etiology of IBD is not fully understood, there is a consensus that loss of homeostasis in the gut microbiota and host immune system plays an important role in the pathogenesis of IBD, where microbiota-derived metabolites act as key factors in host-microbe interactions [30,31,32]. Indeed, high-field NMR-based metabolomics studies have detected variations in the metabolic profiles of feces, urine, plasma, serum, and mucosal biopsies of IBD patients as well as animal models [33,34,35,36,37,38].

As a demonstration experiment of metabolome analysis using benchtop NMR on fecal samples, we conducted a study using a mouse model of DSS-induced colitis, the most widely used model of IBD. We applied both conventional high-field superconducting NMR systems and low-field benchtop NMR to identify and characterize the metabolic profiles of fecal samples from healthy and DSS-induced mice and compared the obtained data. To the best of our knowledge, this is the first study in the field to examine the potential of benchtop NMR for measuring fecal samples.

## 2. Experimental Design

### 2.1. Animals and Sample Collection

The animal experiments were approved by the Institutional Animal Care and Use Committee of the National University Corporation at Hokkaido University and were carried out in accordance with the Hokkaido University Regulations of Animal Experimentation. Six male C57BL/6JJcl mice aged 11 weeks were purchased from CLEA Japan (Tokyo, Japan) and propagated at Hokkaido University. Mice were randomly divided into two groups: the control group and the DSS-induced group (*n* = 3 per group). All mice were housed in different cages for 7 days and cage bedding was changed daily to avoid contamination. The same diet was given to all mice, while 3.5% DSS (molecular weight = 5–1400 kDa) was added to the drinking water of mice in the DSS group to induce colitis. The mice were fasted at the end of day 6 and sacrificed on day 7. Body weight was measured daily, and the fecal samples were collected at 8 a.m. each day, then frozen at −80 °C. After sample collection, the fecal samples were lyophilized and pulverized, and approximately 500–800 mg of feces powder was obtained and stored at −30 °C until the NMR measurement. Colonic tissue was harvested, fixed, sliced, and stained with hematoxylin and eosin (H&E) for histological analysis.

### 2.2. Fecal Sample Processing and ^1^H NMR Measurement

For the mouse fecal samples collected from day 0 to day 5 (*n* = 36), approximately 250–300 mg powdered feces were weighed and mixed with a 1:4 (*w*/*v*) ratio of phosphate buffer (50 mM sodium phosphate, pH 7.4) containing 0.004% sodium azide (NaN_3_) and 10% D_2_O (99.9 atom % D) with 0.5 mM 3-(trimethylsilyl) propionic-2,2,3,3-d4 acid (TSP) and 1 mM formate as internal standards. The mixture was shaken for 15 min, followed by centrifugation at 15,000 rpm for 10 min at 4 °C. The supernatant was collected afterward, and ultrafiltration was conducted overnight using a 5 kDa cut-off centrifugal filter (HMT, Yamagata, Japan) at 9100× *g* and 4 °C. Then, 550 μL of the filtrate was transferred to a 5.0 mm NMR tube for both high-field and low-field NMR measurements. In addition, one concentrated sample was prepared with 1000 mg of feces powder from a healthy mouse and extracted with a 1:10 (*w*/*v*) ratio of ultrapure water. After extraction and centrifugation, the supernatant was processed for lyophilization, and the obtained powder was mixed with 550 μL of phosphate buffer containing 0.5 mM TSP.

Low-field NMR measurements were conducted using a Magritek Spinsolve 60 MHz NMR spectrometer (Magritek, Wellington, New Zealand), which is equipped with a 20-slot autosampler carousel without cooling and combined with a Spinsolve Ultra system for high magnetic-field homogeneity and solvent suppression performance. The samples were placed at room temperature (298 K) before measurement. All 1D ^1^H NMR spectra were acquired using a 1D PRESAT pulse sequence with a SAT power (dB) of −65 and SAT period of 3 s for efficient water suppression and a minimal level of loss of signal intensity. The other measurement parameters were as follows: 128 scans, sweep width of 81 ppm, time-domain size of 32,768, acquisition time of 3.2 s, and a repetition time of 7 s (acquisition + relaxation). The temperature of the magnet was controlled at 299.65 K. In addition, QuickShim was performed at the interval of each measurement using a standard shim sample containing 5% H_2_O and 95% D_2_O.

For high-field NMR measurements, ^1^H NMR spectra were recorded on a Bruker AVANCE Neo 800 MHz spectrometer (Bruker Biospin, Rheinstetten, Germany) equipped with a 5 mm TCI (N) H&F cryoprobe with a Z-gradient at 298 K and an autosampler (SampleJet). To maintain the same measurement parameters as the benchtop NMR spectroscopy, a simple presaturation pulse sequence (zgpr) was applied to all samples with 128 scans, sweep width of 12 ppm, time domain size of 65,536, acquisition time of 3.4 s, relaxation time of 3.6 s and mixing time of 100 ms. In addition, a 1D noesy pulse sequence with water presaturation (noesypr1d) was also applied for quantitative analysis with an acquisition time of 3.4 s and relaxation time of 1.6 s for quantitative targeted analysis.

### 2.3. Data Analysis

All free induction decays (FIDs) measured by both 60 MHz and 800 MHz spectrometers were multiplied with an exponential line broadening function (sexp) of 0.2 Hz prior to Fourier transformation. Then the obtained ^1^H NMR spectra were manually corrected for phase and baseline distortion, and the chemical shift was referenced to TSP at δ = 0.0 ppm using Delta 5.3 (JEOL, Tokyo, Japan). The spectra were normalized to the peak area of the TSP using Chenomx Processor 8.5 (Chenomx, Edmonton, Canada). The binning sheet was then exported using the total area of the spectral line of 0~10 ppm with a binning value of 0.04 and excluded regions of residual water area (δ = 4.5~5.0 ppm) using the Chenomx Profiler 8.5. For non-targeted multivariate analysis, the data matrix of binning based on the 60 MHz and 800 MHz spectra was imported into SIMCA 15.0 (Umetrics, Umeå, Sweden), followed by Pareto scaling. Principal component analysis (PCA), an unsupervised method, was applied to visualize the clustering and separation within the dataset on the score plots, and the loading plots showed the corresponding contribution of each binning to these distributions. Next, orthogonal partial least squares discriminant analysis (OPLS-DA), a supervised pattern recognition approach, was performed to strengthen the discriminant ability of the model and identify the significant variables contributing to the separation. The quality of the prediction models was assessed by the R2X, R2Y, and Q2 values, which describe the goodness of fit in the X (R2X) and Y (R2Y) variables and predictability, respectively [33]. In addition, the identification and quantitation of metabolites were implemented by the Chenomx Profiler 8.5 based on the Chenomx 800 MHz Database, using noesypr1d program-measured data. Subsequently, the concentrations of the metabolites were unit variance (UV)-scaled, followed by PCA and OPLS-DA. In addition, quantitation of the selected metabolite based on 60 MHz data was performed using three strategies: (1) TSP-normalized integration method by manual selection of certain chemical shift region on Mnova 14.2 (Mestrelab Research, Santiago de Compostela, Spain) and defined as “INT (Region) method”; (2) using Mnova 14.2, the “Generalized Lorentzian” (GL) peak shape was fitted to the spectral line with manual modification of the Lorentzian and Gaussian parameters, followed by TSP-normalized integration for the GL peak and defined as “curve fitting (Mnova)”; (3) the standard solution was used as a spectral reference to create and optimize the in-house 60 MHz library in Chenomx Spin Simulator, then the peak shape was pre-defined by the signal of TSP, followed by curve fitting to minimize the subtraction line based on the in-house prepared database using the Chenomx Profiler and defined as “curve fitting (Chenomx) method”.

Statistical analyses were performed using GraphPad Prism 8 (GraphPad Software, San Diego, CA, USA). Student’s *t*-test and one-way ANOVA with Tukey’s post hoc test were used to compare the concentrations between the groups and the accuracy of quantification using different methods, respectively. Statistical significance was set at *p* < 0.05.

## 3. Results

### 3.1. Histological Assessment

The DSS-induced colitis model was successfully constructed, as indicated by the significantly decreased body weight between days 5 and 6, as well as a shorter length of the large intestine (Figure S1A,B). In addition, H&E staining of the colon sections demonstrated epithelial erosion and ulceration, loss of goblet cells and mucus layer, and immune cell infiltration after 7 days of induction (Figure S1C), indicating severe colon inflammation in the DSS-induced mice.

### 3.2. NMR Spectra of Mouse Feces Acquired on 60 MHz and 800 MHz and Metabolites Assignment

**Figure 1:**
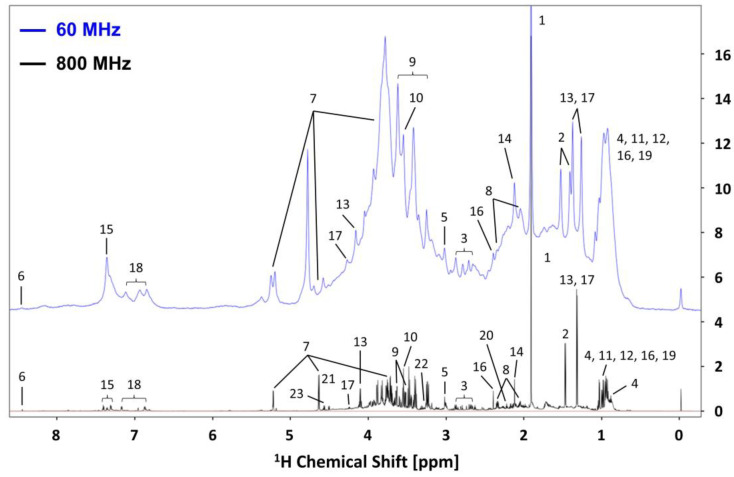
^1^H NMR spectra of the same mouse feces obtained by 60 MHz (blue) and 800 MHz (black) spectroscopy. The vertical axis of the 60 MHz spectrum was expanded to make the peaks easier to recognize, so the intensities of the peaks from the same compound in the two spectra were different. Annotation: 1. Acetate; 2. Alanine; 3. Aspartate; 4. Butyrate; 5. Creatine; 6. Formate; 7. Glucose; 8. Glutamate; 9. Glycerol; 10. Glycine; 11. Isoleucine; 12. Leucine; 13. Lactate; 14. Methionine; 15. Phenylalanine; 16. Propionate; 17. Threonine; 18. Tyrosine; 19. Valine; 20. 5-Aminopentanoate; 21. Arabinose; 22. Taurine; 23. Xylose. The other metabolites include: 2-Hydroxyisovalerate; 3-Hydroxybutyrate; 3-Methyl-2-oxovalerate; 4-Hydroxybenzoate; Choline; Dimethylamine; Ethanol; Fucose; Fumarate; Galactose; Glutamine; Isobutyrate; Isovalerate; Methanol; Nicotinate; Sarcosine; Trimethylamine; and UMP.

Figure 1 shows representative ^1^H NMR spectra of the same highly concentrated fecal sample from healthy C57BL/6JJcl mice measured by both 60 MHz and 800 MHz NMR spectrometers, which represent the best conditions for fecal sample extraction to identify as many compounds as possible. Then, forty-one metabolites were identified in the 800 MHz spectrum based on the Chenomx database and referring to the published literature [39,40]. Despite the low sensitivity of low-field NMR, the same 128 scans used in the 800 MHz NMR system were sufficient to detect the peaks of metabolites in the sample with a good signal-to-noise ratio. Although low-field NMR has problems with signal overlap owing to its low resolution, metabolites with prominently higher concentrations (e.g., singlet resonance derived from acetate at 1.92 ppm), less complex signal patterns (e.g., doublet resonance derived from alanine at 1.48 ppm), and isolated regions (e.g., multiplet resonance derived from tyrosine at 6.95 ppm) were identified without ambiguity. It is worth noting that for a given compound, J-coupling and peak-integrated intensity are independent of magnetic field strength, resulting in different peak positions due to signal splitting in high-field and low-field NMR spectra, and thus different overall spectral patterns (see the example of a pure alanine and isoleucine sample in Figure S2). We finally succeeded in the assignment of 19 metabolites (annotation no.1–19) to the 60 MHz spectra by referring to the assignment of 800 MHz spectra and their corresponding J values, including amino acids, short-chain fatty acids (SCFAs), creatine, formate, glucose, glycerol and lactate. However, the branched-chain amino acids (BCAAs), propionate and butyrate located at 0.7~1.1 ppm could not be clearly distinguished in the 60 MHz NMR spectra owing to the high degree of congestion, although the concentrations of these metabolites were considered relatively high.

### 3.3. Multivariate Analysis Characterized Metabolomic Profiling of Mouse Feces Acquired on 60 MHz and 800 MHz NMR Spectrometers

To determine whether the metabolomics analysis based on 60 MHz NMR spectra was performed effectively to discriminate between the control group and DSS-induced group, and provided comparable results with 800 MHz, multivariate analysis was performed for both 60 MHz and 800 MHz NMR spectra. When PCA was conducted on data from days 0 to 5 acquired at 60 MHz (Figure S3A; PC1 = 36.4%, PC2 = 23.5%), the DSS-induced group on days 3–5 showed a cluster separated from the others, whereas the DSS-induced group on days 0–2 was mixed with the control group. The PCA score plot based on 800 MHz spectral data (Figure S3C; PC1 = 40.5%, PC2 = 23.6%) also showed a similar separation tendency to that of 60 MHz. Then, PCA was performed on the data excluding days 0–2 to characterize the spectral changes caused by the development of DSS-induced colitis and to facilitate a comparison of the 60 MHz and 800 MHz results.

**Figure 2:**
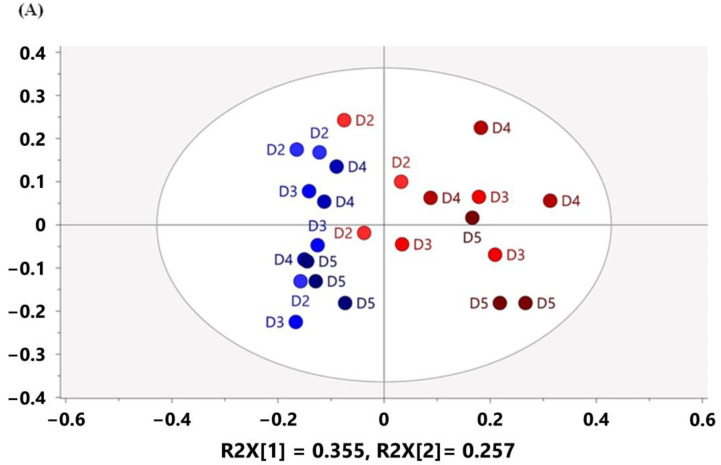

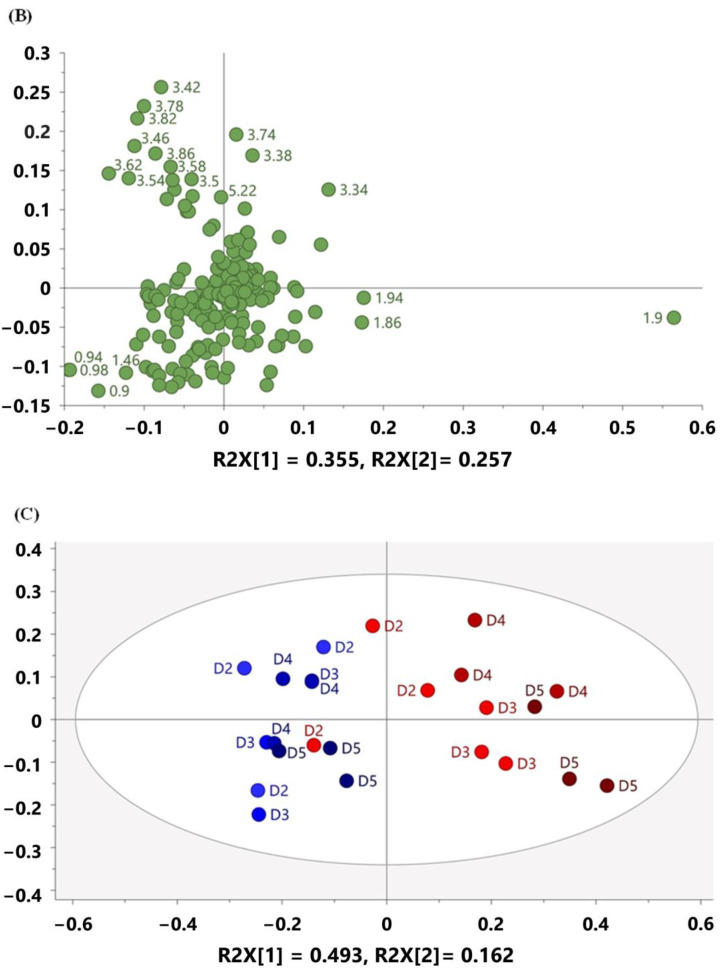

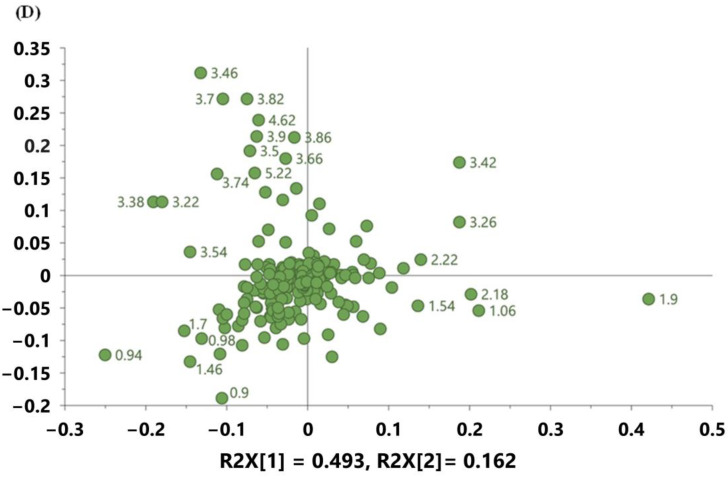
(**A**) PCA score plot of mouse feces in the control group (blue) and DSS group (red) from day 2 to day 5, acquired on the 60 MHz NMR spectrometer, PC1 = 35.5%, PC2 = 25.7%; (**B**) loading plot of (**A**); (**C**) PCA score plot of mouse feces in the control group (blue) and DSS group (red) from day 2 to day 5, acquired on the 800 MHz NMR spectrometer, PC1 = 49.3%, PC2 = 16.2%; (**D**) loading plot of (**C**). The depth of the color in the score plots increased as the cultivation time progressed. R2X[1] and R2X[2] represent the first principal component and the second principal component, respectively.

In the 60 MHz data from days 2 to 5 (Figure 2A; PC1 = 35.5%, PC2 = 25.7%), the shift and separation of the DSS-induced group from the control group became obvious, as it is notable that the separation of the two groups started on day 2 and completely separated from day 3 along PC1. According to the PCA loading plot (Figure 2B), the 1.9 ppm signal contributed positively and predominantly to PC1, suggesting higher concentrations in the DSS-induced group. On the contrary, 0.9~1.0 ppm and 3.4~3.9 ppm showed negative contributions. Importantly, the PCA based on 800 MHz data (Figure 2C; PC1 = 49.3%, PC2 = 16.2%) also showed a shift in the metabolic signature of DSS-induced mice on the score plot, as well as the positive contribution of 1.9 ppm and negative contribution of 0.9~1.0 ppm and 3.4~3.9 ppm (Figure 2D). In addition, it can be noticed that positive contribution of 3.26 and 3.42 ppm and negative contribution of 3.22 and 3.38 ppm to PC1 were demonstrated only in the PCA loading plot based on 800 MHz.

**Figure 3:**
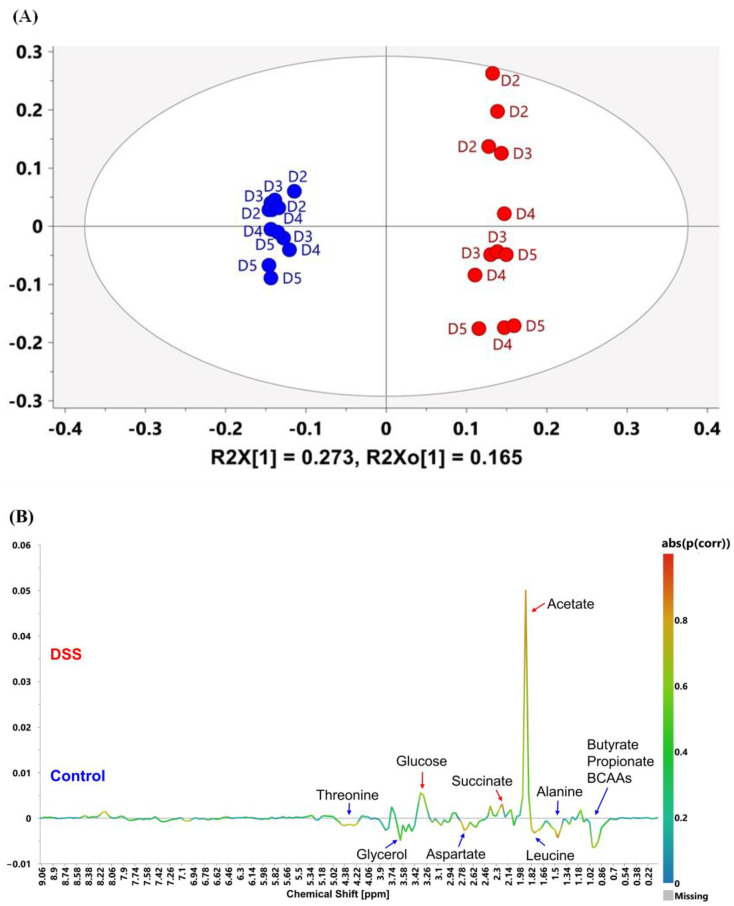
(**A**) OPLS-DA score plot of mouse feces in the control group (blue) and DSS group (red) from day 2 to day 5, acquired on the 60 MHz NMR spectrometer; (**B**) OPLS coefficient plot (S-line) of (**A**). The top end with positive values illustrates the increased relative intensity of bins with DSS treatment, while that with negative values represent the decreased relative intensity in the DSS group. The color is associated with the significance of variables in classifying the groups as shown on the right side of the plot, where the absolute value of the correlation coefficients is shown.

Subsequently, OPLS-DA was used to examine the discriminant ability and improve the interpretability of the model acquired at 60 MHz. All data were integrated into two groups (control and DSS-induction), and information on cultivation time was eliminated prior to constructing the OPLS-DA model. Figure 3A shows the OPLS-DA score plot of mouse feces on days 2–5 in the control and DSS-induced groups. The R2X, R2Y, and Q2 values of this model were 0.698, 0.992, and 0.927, respectively, suggesting a statistical significance for the separation of the two groups. The discriminators of the two groups were summarized by combining the OPLS coefficient plot (S-line, Figure 3B) and the Variable Importance for the Projection (VIP) plot, in which the VIP values were larger than 1. According to Figure 3B, the DSS-induced group had a higher intensity at 1.9 ppm, 2.22 ppm, 2.38 ppm, and 3.26~3.34 ppm; and a lower intensity at 0.9~0.98 ppm, 1.42~1.5 ppm, 1.74~1.82 ppm, 2.7~2.78 ppm, 3.54~3.62 ppm, and 4.22~4.42 ppm. Interpreting these signal contributions on the basis of metabolite assignment in the 60 MHz spectra (Figure 1) suggested that concentrations of butyrate (0.9~0.98 ppm), propionate (0.9~0.98 ppm), isoleucine (0.9~0.98 ppm), valine (0.9~0.98 ppm), leucine (0.9~0.98 ppm; 1.74~1.82 ppm), alanine (1.42~1.5 ppm), aspartate (2.7~2.78 ppm), glycerol (3.54~3.62 ppm) and threonine (4.22~4.42 ppm) were likely decreased in the DSS-induced group compared to the control group. On the other hand, the concentrations of acetate (1.9 ppm), succinate (2.38 ppm), and glucose (3.26~3.34 ppm) were likely increased by DSS treatment. For comparison, the OPLS-DA score plot and OPLS coefficients based on the binning of 800 MHz NMR spectra (Figure S4) showed similar results to the 60 MHz data. However, differences in some points were observed in the 800 MHz S-line data (Figure S4B). An increased intensity of taurine (3.26, 3.42 ppm), which could not be isolated or identified at 60 MHz, was observed. In addition, the 800 MHz data gave the opposite contribution of propionate (1.06 ppm) and glucose (3.22, 3.38, 3.46 ppm) to that of 60 MHz, which was likely due to overlap with the other signals. Adjusting binning values did not essentially change the results. Furthermore, these alterations in metabolites were verified by quantitative targeted analysis based on the 800 MHz platform (Figure S5).

### 3.4. Potential of 60 MHz Benchtop NMR for Quantitative Analysis

A higher intensity of 1.9 ppm, which was assigned to the resonance of acetate, has been characterized as the most important feature in the NMR spectra acquired from the fecal samples of DSS-induced mice. The high concentration of the acetate compared to other metabolites and its singlet peak made it easily observable. Thus, we expected to quantify the concentration of acetate as a key biomarker in our model to discriminate between the two groups and substantiate the potential of 60 MHz benchtop NMR for further quantification of metabolites.

First, a series of sodium acetate pure samples (concentration = 2–20 mM) were prepared and measured by both 60 MHz and 800 NMR spectrometers three times to obtain the calibration curves (Figure S6). The calibration curve was prepared using three methods: (1) INT (Region): simple TSP-normalized integration of a chemical shift region; (2) Curve fitting (Mnova): TSP-normalized integration for the “Generalized Lorentzian” (GL) shaped peak; and (3) Curve fitting (Chenomx): curve fitting using in-house prepared 60 MHz database or the Chenomx built-in 800 MHz database. It was confirmed that all methods showed good linearity and reproducibility (r^2^ > 0.999) at both 60 MHz and 800 MHz.

**Figure 4:**
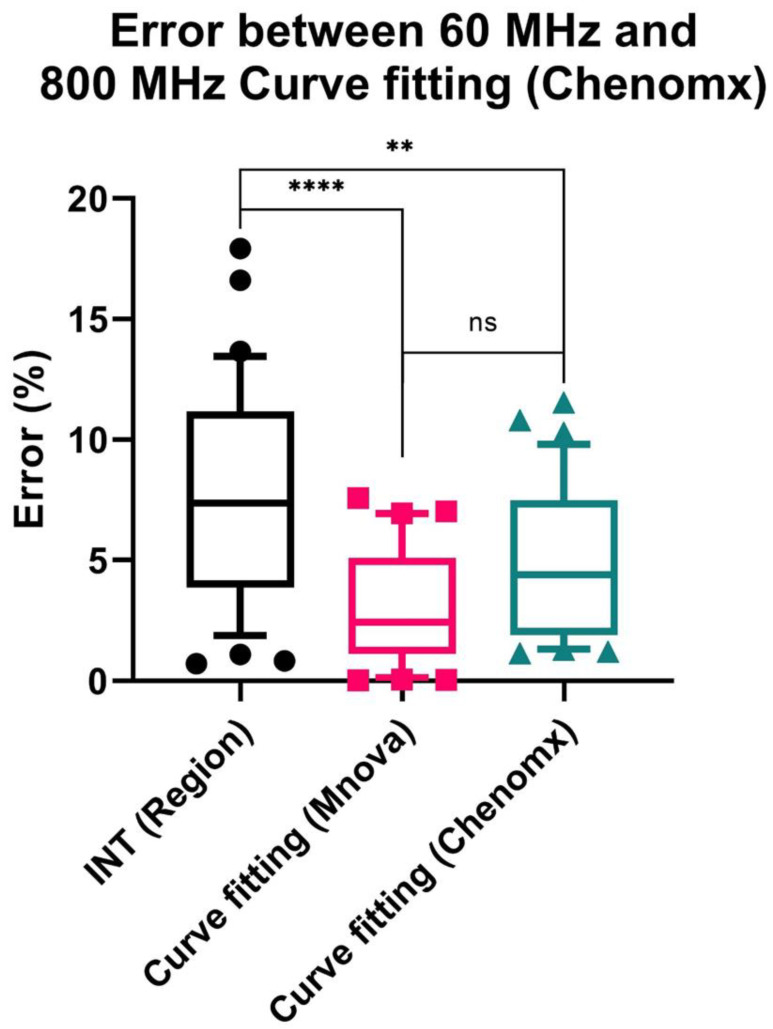
Box plot of the percentage error between the concentration of acetate quantified by each method and the reference (the concentration quantified by curve fitting method based on the original 800 MHz Chenomx database). INT (Region): TSP-normalized integration method by manual selection of chemical shift region; Curve fitting (Mnova): the “Generalized Lorentzian” (GL) peak shape was fitted to the spectral line and modified, followed by TSP-normalized integration for the GL peak using the Mnova; Curve fitting (Chenomx): the peak shape was pre-defined by the signal of TSP, followed by manual fitting by referring to the in-house prepared 60 MHz database or the Chenomx built-in 800 MHz database. The upper and lower whiskers represent the 90th and 10th percentile, respectively. One-way ANOVA with Tukey’s post hoc test was used for the comparison. ****: *p* < 0.0001, **: *p* < 0.01, ns: not significant.

Subsequently, the concentrations of acetate in mouse fecal samples based on 60 MHz and 800 MHz spectra were quantified using these methods and compared with the quantification result using the 800 MHz Chenomx database, which is considered as the reference in the present study (Tables S1 and S2). Additionally, paired differences were calculated, followed by the mean, standard deviation, standard error, 95% confidence interval (CI) of the differences and mean absolute error (MAE) (Table S3) to examine the accuracy and reproducibility of 60 MHz data based on each method, and then summarized by absolute percentage error (Figure 4). In the case of quantification of 800 MHz spectra, all methods showed good reproducibility, suggesting that the systematic error was small between each method (Table S2). However, the simple INT (Region) method for 60 MHz showed a larger difference from the reference concentration, with an MAE of 0.751 mM. In contrast, quantification using curve fitting (Mnova and Chenomx) demonstrated lower MAE (0.316 and 0.484 mM, respectively) and a narrow 95% CI of difference (−0.096–0.191 and −0.395–−0.016 mM, respectively). No significant difference was detected between the two curve fitting methods, although the curve fitting (Mnova) showed a relatively lower MAE value.

**Figure 5:**
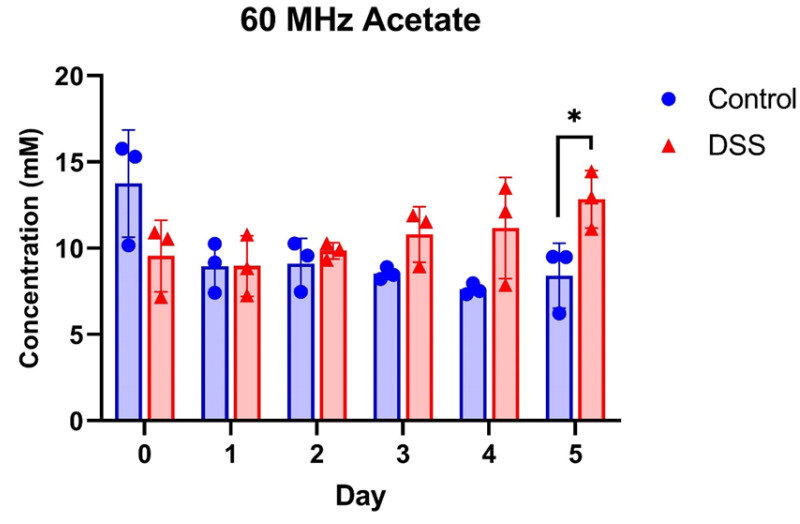
The concentration of acetate in mouse fecal samples quantified by 60 MHz spectra using the “Curve fitting (Mnova)” method. Welch’s unequal variances *t*-test was used for the comparison. *: *p* < 0.05.

The concentration of acetate quantified by the curve fitting (Mnova) method using 60 MHz spectra in the control mice and DSS-induced mice at each time point is depicted in Figure 5. As shown in Figure 5, the DSS-induced group had a higher level of acetate than the control group from days 3 to 5, and the concentration of acetate in the DSS-induced group was significantly higher at day 5, which was consistent with 800 MHz data using the curve fitting (Chenomx) method (Figure S7).

## 4. Discussion

NMR spectrometry with an operating frequency of 600 MHz or higher has been one of the most frequently used research techniques applied for non-targeted and targeted metabolomic analyses or their combinations to screen key biomarkers underlying the pathogenesis of diseases such as IBD [33,41]. However, further applications of NMR-based metabolomics for point-of-care diagnosis and monitoring are restricted by their large size, high cost, and operational difficulties [21]. In this study, for the first time, we performed a low-field, benchtop NMR-based metabolomic analysis of fecal samples to characterize the modified metabolic profile of DSS-induced colitis model mice compared to healthy mice.

For the concentrated fecal extracts of healthy mice, we identified 19 metabolites in the 60 MHz NMR spectra despite signal boarding and overlapping, presenting an attractive result that the benchtop NMR platform would provide informative metabolomics data. These assigned metabolites included amino acids (e.g., branched-chain amino acids, alanine, and tyrosine), SCFAs (acetate, propionate, and butyrate), creatine, formate, glucose, glycerol, and lactate. More importantly, non-targeted multivariate analyses including PCA and OPLS-DA illustrated the separation of the DSS-induced group and the control group from day 2 to day 5, indicating the discriminant ability and feasibility of benchtop NMR for metabolomics studies of inflammatory bowel diseases. These results are highly comparable to the metabolic signature profiled by the high-field NMR platform, which is consistent with previous urinary metabolomics studies [21,24]. Notably, the alteration of the metabolic profile of DSS-treated mice (from day 2) occurred earlier than the onset of significant weight loss resulting from stool bleeding, wasting and diarrhea, which are considered the primary clinical symptoms of IBD pathogenesis in both experimental animal models and humans [42,43,44]. Compared with the current standard diagnosis based on endoscopic, histological, and radiologic techniques [33,35], the low-field, benchtop NMR might be a potential tool for noninvasive early diagnosis of IBD. Furthermore, the metabolomics data of mouse feces were acquired without complex sample processing, thus enabling quick and easy measurement of various samples, which may shorten the duration between detection and diagnosis at point-of-care sites [24]. Similarly, in the field of food processing and quality control, research to simplify the preparation of samples for measurement is indispensable [14,16]. However, it is essential to verify its feasibility through large-scale human studies using several samples in the future considering the variability and individual difference of human beings, and the protocol of sample processing needs to be validated and standardized. Furthermore, to facilitate such studies with even more samples, one of the issues may be to promote the introduction of autosamplers that can handle more samples, equipped with cooling systems in benchtop NMR.

The alteration of metabolites derived from both 60 MHz and 800 MHz NMR measurements demonstrated elevated levels of acetate and succinate in the feces of DSS-treated mice. In contrast, they were also characterized by lower concentrations of butyrate, branched-chain amino acids, alanine, aspartate, threonine, and glycerol. In IBD patients and colitis model mice, metabolomics and metagenomics studies have reported dysbiosis of the gastrointestinal microbiota and microbial metabolism [45,46,47]. SCFAs, mainly acetate, propionate and butyrate, are carboxylic acids produced by the microbial fermentation of polysaccharides. SCFAs are important metabolites in maintaining intestinal homeostasis, strengthening gut barrier function, supplying energy for colonic epithelial cells, and acting as signaling molecules [48]. In particular, it has been documented that the abundance of SCFA-producing bacteria such as the genera *Roseburia* and *Faecalibacterium*, as well as the concentration of SCFAs, especially butyrate, were reduced in fecal samples of IBD patients [35,44,49,50,51]. This is consistent with the reduction in butyrate levels observed in the DSS-treated group in our experiment. Remarkably, our study showed a predominantly increased amount of acetate in DSS-induced mice compared to that in the control. It has been suggested that only ~5% of SCFAs produced by bacteria remain in feces, and alterations in transit, absorption and utilization might lead to different directions of change in the content of SCFAs [32]. Thus, the significantly increased concentration of acetate in DSS-treated mouse feces may be attributed to the defective uptake of energy by the colonic epithelium. In addition, elevated concentrations of BCAAs, alanine, and lysine in the feces of IBD patients have been reported by Marchesi et al. and Bjerrum et al. [35,52]. Correspondingly, the change in the metabolic profile of colonic tissues was characterized by lower levels of these amino acids [37,53], indicating malabsorption under inflammatory conditions [54]. However, metabolomic research using mouse models might lead to diverse fluctuations, and the tendency was dependent on each study [41,55]. Therefore, the relatively lower levels of amino acids in DSS mouse feces are likely due to decreases in dietary protein degradation or amino acid biosynthesis caused by reduced bacterial populations [55]. Moreover, succinate, a tricarboxylic acid cycle intermediate, acts as an important pro-inflammatory signal in the host [32], and a previous study demonstrated an increased level of succinate in DSS-induced mice [41].

The signal overlapping problems caused by adjacent chemical shifts appear to be the main limitation for the identification and quantification of metabolites in biological samples using low-field, benchtop NMR, and particular attention should be paid when analyzing and interpreting metabolic profiles, as suggested by previous studies [14,22]. Some compounds have significantly different signal patterns in low-field and high-field NMR spectra due to the effect of J-coupling splitting. Although it is expected that such differences can be reduced by spectral binning, they will inevitably affect the analysis. Indeed, multivariate analyses of 60 MHz data of mouse feces showed misleading information about alterations of propionate, glucose, and taurine, suggesting the limitation of 60 MHz NMR when interpreting the change in metabolic profile in the crowded region at 0.9~1.1 ppm and 3–4 ppm. For example, the resonance of the -CH_3_ group of propionate (t, δ = 1.05 ppm) has a J value of 7.70 Hz, the range of this peak area will occupy 3 × 7.70 Hz/800 Hz = 0.029 ppm when measured by 800 MHz, while the occupied region would be 3 × 7.70 Hz/60 Hz = 0.385 ppm when measured by a 60 MHz NMR spectrometer. Correspondingly, the -C_γ2_H_3_ methyl groups of valine (d, δ = 1.03 ppm) and isoleucine (d, δ = 1.00 ppm) have a J value of 7.05 Hz that would envelop 0.235 ppm. Such signal broadening would lead to an inevitable overlapping problem, and the increased level of propionate would be covered by a decrease in BCAAs. Likewise, the decreased intensity of glucose (δ = 3.22, 3.38, 3.46 ppm) was obscured by elevated concentrations of taurine (δ = 3.26, 3.42 ppm), which was difficult to identify in the 60 MHz spectra.

The performance of benchtop NMR for quantitative analysis has been demonstrated in previous studies, where the concentration of glucose, a common marker of type 2 diabetes, was quantified using the α-glucose anomer C1-H signal at 5.2 ppm in urine samples [21,22]. In this study, we attempted to quantify the concentration of acetic acid in mouse fecal samples by low-field benchtop NMR spectroscopy using three quantitative methods. Quantification using simple integral intensities of the 60 MHz spectra resulted in a fairly large error due to signal overlap, which was greatly reduced by curve fitting methods. In addition, the quantitative results of this study showed a slightly smaller error trend for the Mnova software than for the Chenomx software, which is commonly used for NMR metabolome analysis, although the difference was not significant. This may be because more fitting parameters of the generalized Lorentzian were adjusted in the curve fitting by Mnova in this study. In addition, it has been suggested that the concentrations of compounds with simple resonance patterns, such as acetate, alanine, BCAAs, lactate, citrate and succinate, are suitable for quantitative analyses [14]. However, the spectra of fecal samples suffer from severe overlapping and spectral background effects. As a result, it remains challenging to quantify metabolites other than acetate in fecal samples using simple integration or curve fitting methods, which appears to be a limitation of our study.

The limitation from the aspect of identification and quantification of benchtop NMR would be broken out with the progress of both hardware and methodologies of data analysis. For example, stronger permanent magnets have been applied in a novel SpinSolve 90 MHz NMR spectrometer (Magritek). In addition, with the increasing accessibility of massive NMR datasets and the development of algorithms, deep learning methods such as image recognition and image labeling neural networks have shown their potential for fast processing and prediction of the spectra and deconvolution of the peak overlap [56,57]. Furthermore, field-invariant methods based on the quantum mechanical properties of spin systems have been attempted to enhance the quantitative analysis of benchtop NMR [19,58,59].

In summary, we presented the potential applications of low-field benchtop NMR for the rapid diagnosis of IBD using a DSS-induced mouse model. The metabolic profile characterized by 60 MHz data showed good comparability with the 800 MHz data. In addition, although it demonstrated high reproducibility for the quantification of metabolites, it was difficult to carry out a detailed quantitative analysis. Therefore, further exploration of analytical methods, such as machine learning, is needed to deal with the shortcomings of benchtop NMR and realize the application of this technique in the metabolomics field. This pilot study was the first step in a series of possible future studies, including diagnostics using human feces with benchtop NMR.

## Data Availability

The data presented in this study are available on request from the corresponding author. The data are not publicly available due to privacy.

## Supplementary Materials

The following supporting information can be downloaded at: https://www.mdpi.com/article/10.3390/metabo13050611/s1, Figure S1: (A) Body weight; (B) the length of large intestine and small intestine; (C) hematoxylin–eosin (H&E) staining of colon section collected from control mice (upper) and DSS-induced mice (down) at day 7. Scale bars: 50 µm; Figure S2: ^1^H NMR spectra of pure alanine and isoleucine sample obtained by 60 MHz and 800 MHz spectroscopy, respectively. Chemical shift of 0.5–2.5 ppm was shown. Note that the vertical axis of the 60 MHz spectra was expanded to facilitate peak recognition, and direct peak area comparison was not possible; Figure S3: (A) PCA score plot of mice feces of control group (blue) and DSS group (red) from day 0 to day 5 acquired on a 60 MHz NMR spectrometer, PC1 = 36.4%, PC2 = 23.5%; (B) loading plot of Figure S2A; (C) PCA score plot of mice feces of control group (blue) and DSS group (red) from day 0 to day 5 acquired on a 800 MHz NMR spectrometer, PC1 = 40.5%, PC2 = 23.6%; (D) loading plot of Figure S2C. The depth of the color in the score plots increased as the cultivation time progressed. The R2X[1] and R2X[2] represent the first and second principal component, respectively; Figure S4: (A) OPLS-DA score plot of mice feces of control group (blue) and DSS group (red) from day 2 to day 5, acquired on an 800 MHz NMR spectrometer; (B) OPLS coefficient plot (S-line) of Figure S3A. The top end with the positive value illustrates the increased relative intensity of bins with DSS treatment, while the negative value represent the decreased relative intensity in the DSS group. The color is associated with the significance of variables in separating the groups as shown on the right side of the plot, where the absolute value of the correlation coefficients was shown; Figure S5: (A) OPLS-DA score plot of concentration of metabolites quantified by Chenomx Profiler in mice feces of control group (blue) and DSS group (red) from day 2 to day 5 acquired on an 800 MHz NMR spectrometer; (B) loading plot of Figure S4A; Figure S6: Calibration curve of acetate standard sample measured by a 60 MHz (A) and 800 MHz (B) spectrometer. The concentration of acetate was quantified by three methods. INT (Region): TSP-normalized integration method by manual selection of chemical shift region; Curve fitting (Mnova): the “Generalized Lorentzian” (GL) peak shape was fitted to the spectral line and modified, followed by TSP-normalized integration for the GL peak using the Mnova; Curve fitting (Chenomx): the peak shape was pre-defined by the signal of TSP, followed by manual fitting by referring to the in-house prepared 60 MHz database or the Chenomx built-in 800 MHz database; Figure S7: The concentration of acetate in mouse fecal samples quantified by 800 MHz spectra using the “Curve fitting (Chenomx)” method. Welch’s unequal variances *t*-test was used for the comparison. *: *p* < 0.05; Table S1: Comparison of three quantification methods for the concentration of acetate (mM) on 60 MHz NMR spectra; Table S2: Comparison of integration methods for quantifying the concentration of acetate (mM) on 800 MHz NMR spectra; Table S3: Paired differences of each method for quantifying the concentration of acetate (mM) in the mouse fecal samples based on 60 MHz NMR spectra with the routine method (800 MHz curve fitting using Chenomx).

## References

[1] Beckonert O., Keun H.C., Ebbels T.M.D., Bundy J., Holmes E., Lindon J.C., Nicholson J.K. (2007). Metabolic profiling, metabolomic and metabonomic procedures for NMR spectroscopy of urine, plasma, serum and tissue extracts. Nat. Protoc..

[2] Johnson C.H., Ivanisevic J., Siuzdak G. (2016). Metabolomics: Beyond biomarkers and towards mechanisms. Nat. Rev. Mol. Cell Biol..

[3] Ussher J.R., Elmariah S., Gerszten R.E., Dyck J.R. (2016). The emerging role of metabolomics in the diagnosis and prognosis of cardiovascular disease. J. Am. Coll. Cardiol..

[4] He X., Ji G., Jia W., Li H. (2016). Gut microbiota and nonalcoholic fatty liver disease: Insights on mechanism and application of metabolomics. Int. J. Mol. Sci..

[5] Holmes E., Wist J., Masuda R., Lodge S., Nitschke P., Kimhofer T., Loo R.L., Begum S., Boughton B., Yang R. (2021). Incomplete systemic recovery and metabolic phenoreversion in post-acute-phase nonhospitalized COVID-19 patients: Implications for assessment of Post-Acute COVID-19 Syndrome. J. Proteome Res..

[6] Shao Y., Le W. (2019). Recent advances and perspectives of metabolomics-based investigations in Parkinson’s Disease. Mol. Neurodegener..

[7] Louis P., Hold G.L., Flint H.J. (2014). The gut microbiota, bacterial metabolites and colorectal cancer. Nat. Rev. Microbiol..

[8] Buergel T., Steinfeldt J., Ruyoga G., Pietzner M., Bizzarri D., Vojinovic D., Upmeier zu Belzen J., Loock L., Kittner P., Christmann L. (2022). Metabolomic profiles predict individual multidisease outcomes. Nat. Med..

[9] Deda O., Gika H.G., Wilson I.D., Theodoridis G.A. (2015). An overview of fecal sample preparation for global metabolic profiling. J. Pharm. Biomed. Anal..

[10] Takis P.G., Ghini V., Tenori L., Turano P., Luchinat C. (2019). Uniqueness of the NMR approach to metabolomics. TrAC-Trend Anal. Chem..

[11] Emwas A.H., Roy R., McKay R.T., Tenori L., Saccenti E., Gowda G.A.N., Raftery D., Alahmari F., Jaremko L., Jaremko M. (2019). NMR spectroscopy for metabolomics research. Metabolites.

[12] Bouillaud D., Farjon J., Gonçalves O., Giraudeau P. (2019). Benchtop NMR for the monitoring of bioprocesses. Magn. Reson. Chem..

[13] Zalesskiy S.S., Danieli E., Blümich B., Ananikov V.P. (2014). Miniaturization of NMR systems: Desktop spectrometers, microcoil spectroscopy, and “NMR on a chip” for chemistry, biochemistry, and industry. Chem. Rev..

[14] Grootveld M., Percival B., Gibson M., Osman Y., Edgar M., Molinari M., Mather M.L., Casanova F., Wilson P.B. (2019). Progress in low-field benchtop NMR spectroscopy in chemical and biochemical analysis. Anal. Chim. Acta.

[15] Blümich B., Singh K. (2018). Desktop NMR and its applications from materials science to organic chemistry. Angew. Chem. Int. Ed..

[16] Gouilleux B., Marchand J., Charrier B., Remaud G.S., Giraudeau P. (2018). High-throughput authentication of edible oils with benchtop ultrafast 2D NMR. Food Chem..

[17] Singh K., Blümich B. (2018). Compact low-field NMR spectroscopy and chemometrics: A tool box for quality control of raw rubber. Polymer.

[18] Archambault C.M., Leadbeater N.E. (2016). A benchtop NMR spectrometer as a tool for monitoring mesoscale continuous-flow organic synthesis: Equipment interface and assessment in four organic transformations. RSC Adv..

[19] Matviychuk Y., Haycock S., Rutan T., Holland D.J. (2021). Quantitative analysis of wine and other fermented beverages with benchtop NMR. Anal. Chim. Acta.

[20] Edgar M., Percival B.C., Gibson M., Jafari F., Grootveld M. (2021). Low-field benchtop NMR spectroscopy as a potential non-stationary tool for point-of-care urinary metabolite tracking in diabetic conditions. Diabetes Res. Clin. Pract..

[21] Leenders J., Grootveld M., Percival B., Gibson M., Casanova F., Wilson P.B. (2020). Benchtop low-frequency 60 MHz NMR analysis of urine: A comparative metabolomics investigation. Metabolites.

[22] Percival B.C., Grootveld M., Gibson M., Osman Y., Molinari M., Jafari F., Sahota T., Martin M., Casanova F., Mather M.L. (2018). Low-field, benchtop NMR spectroscopy as a potential tool for point-of-care diagnostics of metabolic conditions: Validation, protocols and computational models. High-Throughput.

[23] Ruiz-Cabello J., Sevilla I.A., Olaizola E., Bezos J., Miguel-Coello A.B., Muñoz-Mendoza M., Beraza M., Garrido J.M., Izquierdo-García J.L. (2022). Benchtop nuclear magnetic resonance-based metabolomic approach for the diagnosis of bovine tuberculosis. Transbound. Emerg. Dis..

[24] Izquierdo-Garcia J.L., Comella-del-Barrio P., Campos-Olivas R., Villar-Hernández R., Prat-Aymerich C., De Souza-Galvão M.L., Jiménez-Fuentes M.A., Ruiz-Manzano J., Stojanovic Z., González A. (2020). Discovery and validation of an NMR-based metabolomic profile in urine as TB biomarker. Sci. Rep..

[25] Cui M., Trimigno A., Aru V., Khakimov B., Engelsen S.B. (2020). Human faecal ^1^H-NMR metabolomics: Evaluation of solvent and sample processing on coverage and reproducibility of signature metabolites. Anal. Chem..

[26] Zhao M., Gönczi L., Lakatos P.L., Burisch J. (2021). The burden of inflammatory bowel disease in Europe in 2020. J. Crohn’s Colitis.

[27] GBD 2017 Inflammatory Bowel Disease Collaborators (2020). The global, regional, and national burden of inflammatory bowel disease in 195 countries and territories, 1990–2017: A systematic analysis for the global burden of disease Study 2017. Lancet Gastroenterol. Hepatol..

[28] Wirtz S., Neurath M.F. (2007). Mouse models of inflammatory bowel disease. Adv. Drug Deliv. Rev..

[29] Martin F.P., Rezzi S., Philippe D., Tornier L., Messlik A., Hölzlwimmer G., Baur P., Quintanilla-Fend L., Loh G., Blaut M. (2009). Metabolic assessment of gradual development of moderate experimental colitis in IL-10 deficient mice. J. Proteome Res..

[30] Agus A., Clément K., Sokol H. (2021). Gut microbiota-derived metabolites as central regulators in metabolic disorders. Gut.

[31] Tang Z.Z., Chen G., Hong Q., Huang S., Smith H.M., Shah R.D., Scholz M., Ferguson J.F. (2019). Multi-omic analysis of the microbiome and metabolome in healthy subjects reveals microbiome-dependent relationships between diet and metabolites. Front. Genet..

[32] Lavelle A., Sokol H. (2020). Gut microbiota-derived metabolites as key actors in inflammatory bowel disease. Nat. Rev. Gastroenterol. Hepatol..

[33] Schicho R., Shaykhutdinov R., Ngo J., Nazyrova A., Schneider C., Panaccione R., Kaplan G.G., Vogel H.J., Storr M. (2012). Quantitative metabolomic profiling of serum, plasma, and urine by (1)H NMR spectroscopy discriminates between patients with inflammatory bowel disease and healthy individuals. J. Proteome Res..

[34] Schicho R., Nazyrova A., Shaykhutdinov R., Duggan G., Vogel H.J., Storr M. (2010). Quantitative metabolomic profiling of serum and urine in DSS-induced ulcerative colitis of mice by (1)H NMR spectroscopy. J. Proteome Res..

[35] Marchesi J.R., Holmes E., Khan F., Kochhar S., Scanlan P., Shanahan F., Wilson I.D., Wang Y. (2007). Rapid and noninvasive metabonomic characterization of inflammatory bowel disease. J. Proteome Res..

[36] Le Gall G., Noor S.O., Ridgway K., Scovell L., Jamieson C., Johnson I.T., Colquhoun I.J., Kemsley E.K., Narbad A. (2011). Metabolomics of fecal extracts detects altered metabolic activity of gut microbiota in ulcerative colitis and irritable bowel syndrome. J. Proteome Res..

[37] Balasubramanian K., Kumar S., Singh R.R., Sharma U., Ahuja V., Makharia G.K., Jagannathan N.R. (2009). Metabolism of the colonic mucosa in patients with inflammatory bowel diseases: An in vitro proton magnetic resonance spectroscopy study. Magn. Reson. Imaging.

[38] Bezabeh T., Somorjai R.L., Smith I.C.P., Nikulin A.E., Dolenko B., Bernstein C.N. (2001). The use of ^1^H magnetic resonance spectroscopy in inflammatory bowel diseases: Distinguishing ulcerative colitis from Crohn’s disease. Am. J. Gastroenterol..

[39] Komatsu Y., Shimizu Y., Yamano M., Kikuchi M., Nakamura K., Ayabe T., Aizawa T. (2020). Disease progression-associated alterations in fecal metabolites in SAMP1/YitFc mice, a Crohn’s disease model. Metabolomics.

[40] Li Y., Xie Z., Gao T., Li L., Chen Y., Xiao D., Liu W., Zou B., Lu B., Tian X. (2019). A holistic view of gallic acid-induced attenuation in colitis based on microbiome-metabolomics analysis. Food Funct..

[41] Osaka T., Moriyama E., Arai S., Date Y., Yagi J., Kikuchi J., Tsuneda S. (2017). Meta-analysis of fecal microbiota and metabolites in experimental colitic mice during the inflammatory and healing phases. Nutrients.

[42] Shiomi Y., Nishiumi S., Ooi M., Hatano N., Shinohara M., Yoshie T., Kondo Y., Furumatsu K., Shiomi H., Kutsumi H. (2011). GCMS-based metabolomic study in mice with colitis induced by dextran sulfate sodium. Inflamm. Bowel Dis..

[43] Eichele D.D., Kharbanda K.K. (2017). Dextran sodium sulfate colitis murine model: An indispensable tool for advancing our understanding of inflammatory bowel diseases pathogenesis. World J. Gastroenterol..

[44] Lloyd-Price J., Arze C., Ananthakrishnan A.N., Schirmer M., Avila-Pacheco J., Poon T.W., Andrews E., Ajami N.J., Bonham K.S., Brislawn C.J. (2019). Multi-omics of the gut microbial ecosystem in inflammatory bowel diseases. Nature.

[45] Franzosa E.A., Sirota-Madi A., Avila-Pacheco J., Fornelos N., Haiser H.J., Reinker S., Vatanen T., Hall A.B., Mallick H., McIver L.J. (2019). Gut microbiome structure and metabolic activity in inflammatory bowel disease. Nat. Microbiol..

[46] Morgan X.C., Tickle T.L., Sokol H., Gevers D., Devaney K.L., Ward D.V., Reyes J.A., Shah S.A., LeLeiko N., Snapper S.B. (2012). Dysfunction of the intestinal microbiome in inflammatory bowel disease and treatment. Genome Biol..

[47] Baur P., Martin F.P., Gruber L., Bosco N., Brahmbhatt V., Collino S., Guy P., Montoliu I., Rozman J., Klingenspor M. (2011). Metabolic phenotyping of the Crohn’s disease-like IBD etiopathology in the TNF (ΔARE/WT) mouse model. J. Proteome Res..

[48] Parada Venegas D.P., De La Fuente M.K., Landskron G., González M.J., Quera R., Dijkstra G., Harmsen H.J.M., Faber K.N., Hermoso M.A. (2019). Short chain fatty acids (SCFAs)-mediated gut epithelial and immune regulation and its relevance for inflammatory bowel diseases. Front. Immunol..

[49] Huda-Faujan N., Abdulamir A.S., Fatimah A.B., Anas O.M., Shuhaimi M., Yazid A.M., Loong Y.Y. (2010). The impact of the level of the intestinal short chain fatty acids in inflammatory bowel disease patients versus healthy subjects. Open Biochem. J..

[50] Takaishi H., Matsuki T., Nakazawa A., Takada T., Kado S., Asahara T., Kamada N., Sakuraba A., Yajima T., Higuchi H. (2008). Imbalance in intestinal microflora constitution could be involved in the pathogenesis of inflammatory bowel disease. Int. J. Med. Microbiol..

[51] Kumari R., Ahuja V., Paul J. (2013). Fluctuations in butyrate-producing bacteria in ulcerative colitis patients of North India. World J. Gastroenterol..

[52] Bjerrum J.T., Wang Y., Hao F., Coskun M., Ludwig C., Günther U., Nielsen O.H. (2015). Metabonomics of human fecal extracts characterize ulcerative colitis, Crohn’s disease and healthy individuals. Metabolomics.

[53] Ooi M., Nishiumi S., Yoshie T., Shiomi Y., Kohashi M., Fukunaga K., Nakamura S., Matsumoto T., Hatano N., Shinohara M. (2011). GC/MS-based profiling of amino acids and TCA cycle-related molecules in ulcerative colitis. Inflamm. Res..

[54] Jansson J., Willing B., Lucio M., Fekete A., Dicksved J., Halfvarson J., Tysk C., Schmitt-Kopplin P. (2009). Metabolomics reveals metabolic biomarkers of Crohn’s disease. PLoS ONE.

[55] Hong Y.S., Ahn Y.T., Park J.C., Lee J.H., Lee H., Huh C.S., Kim D.H., Ryu D.H., Hwang G.S. (2010). ^1^H NMR-based metabonomic assessment of probiotic effects in a colitis mouse model. Arch. Pharm. Res..

[56] Cobas C. (2020). NMR signal Processing, prediction, and structure verification with machine learning techniques. Magn. Reson. Chem..

[57] Li D.W., Hansen A.L., Yuan C., Bruschweiler-Li L., Brüschweiler R. (2021). DEEP picker is a deep neural network for accurate deconvolution of complex two-dimensional NMR spectra. Nat. Commun..

[58] Edgar M., Kuhn S., Page G., Grootveld M. (2022). Computational simulation of ^1^H NMR profiles of complex biofluid analyte mixtures at differential operating frequencies: Applications to low-field benchtop spectra. Magn. Reson. Chem..

[59] Matviychuk Y., Steimers E., von Harbou E., Holland D.J. (2020). Bayesian approach for automated quantitative analysis of benchtop NMR Data. J. Magn. Reson..

