# Supplementary material for: Application of Benchtop NMR for Metabolomics Study Using Feces of Mice with DSS-Induced Colitis
Source: Metabolites. 2023 Apr 28;13(5):611. doi: 10.3390/metabo13050611 (PMC10224185; doi:10.3390/metabo13050611)
Supplement: Supplementary file 1 [file metabolites-13-00611-s001.zip › metabolites-2332521-supplementary.pdf]

(A)

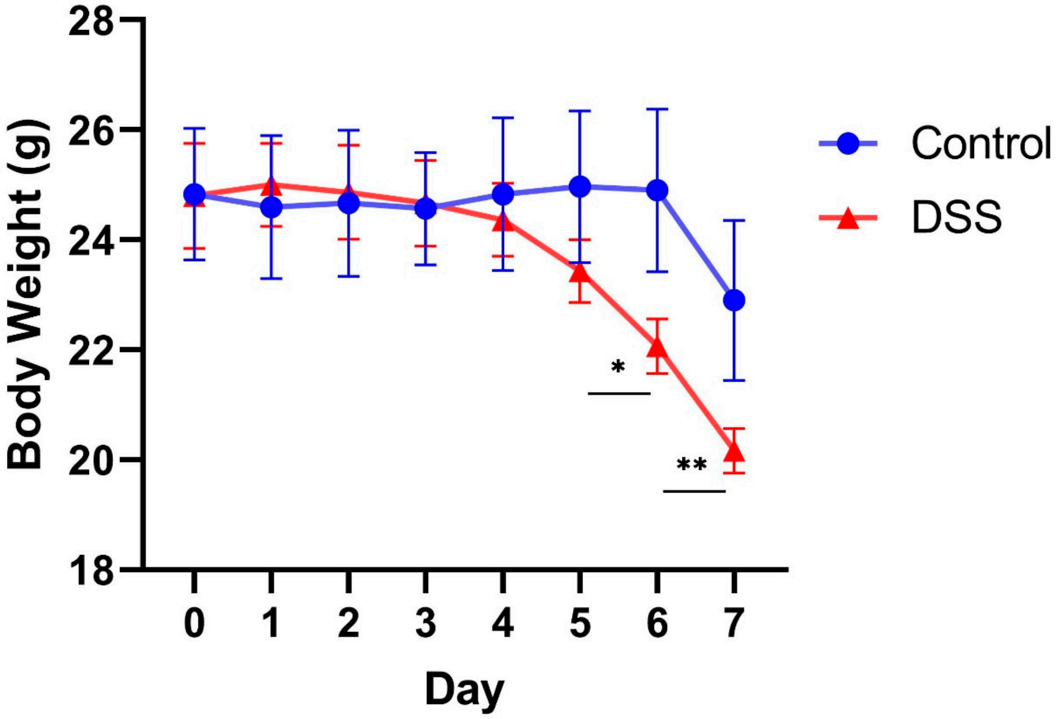

28
26
24
22
20
18
Body Weight (g)
0
1
2
3
4
5
6
7
Day
*
**
Control
DSS

(B)

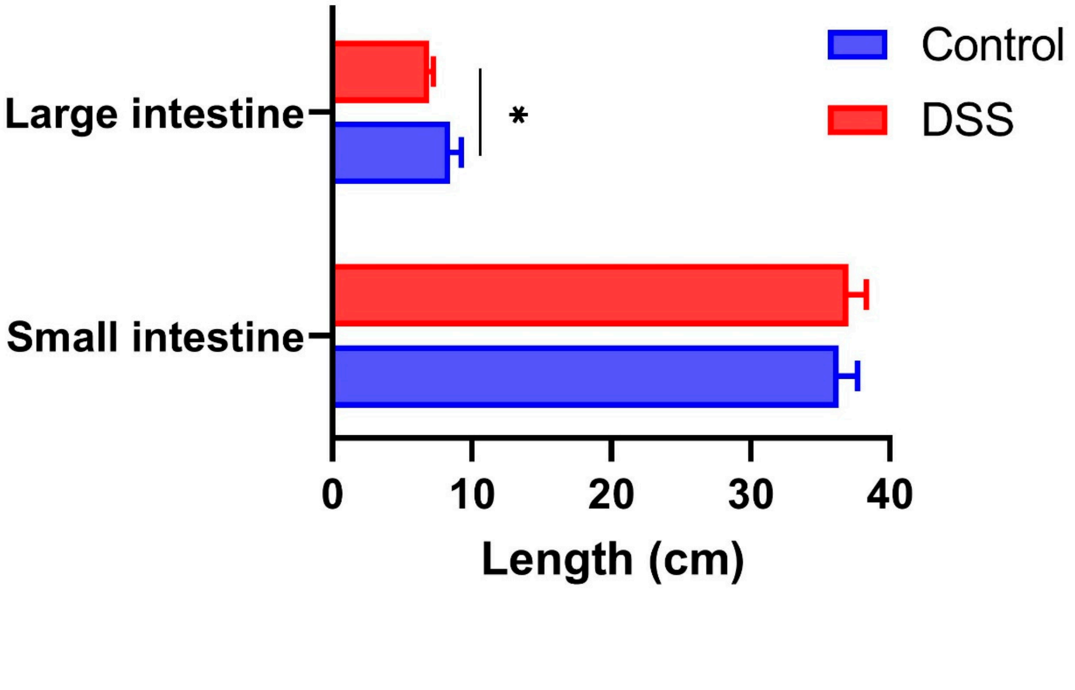

Large intestine
Small intestine
*
0
10
20
30
40
Length (cm)
Control
DSS

(C)

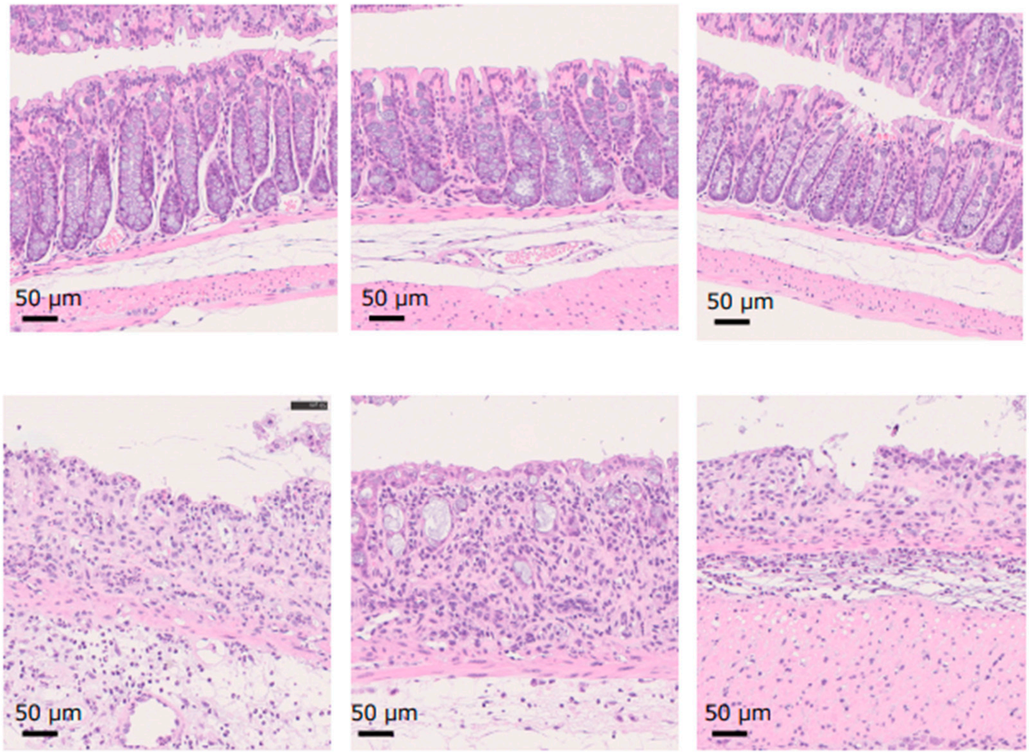

Figure S1. (A) Body weight; (B) the length of large intestine and small intestine; (C) hematoxylin–eosin (H&E) staining of colon section collected from control mice (upper) and DSS-induced mice (down) at day 7. Scale bars: 50 μm

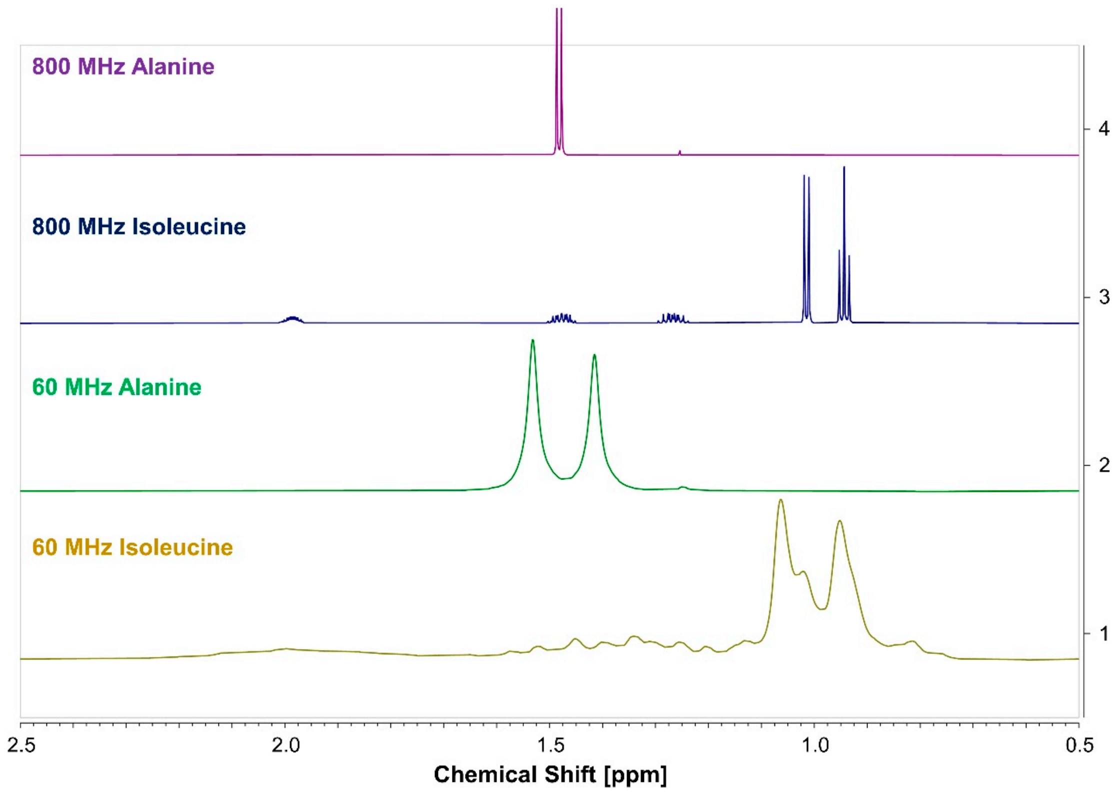

Figure S2. ^{1}H NMR spectra of pure alanine and isoleucine sample obtained by 60 MHz and 800 MHz spectroscopy, respectively. Chemical shift of 0.5-2.5 ppm was shown. Note that the vertical axis of the 60 MHz spectra was expanded to facilitate peak recognition, and direct peak area comparison was not possible.

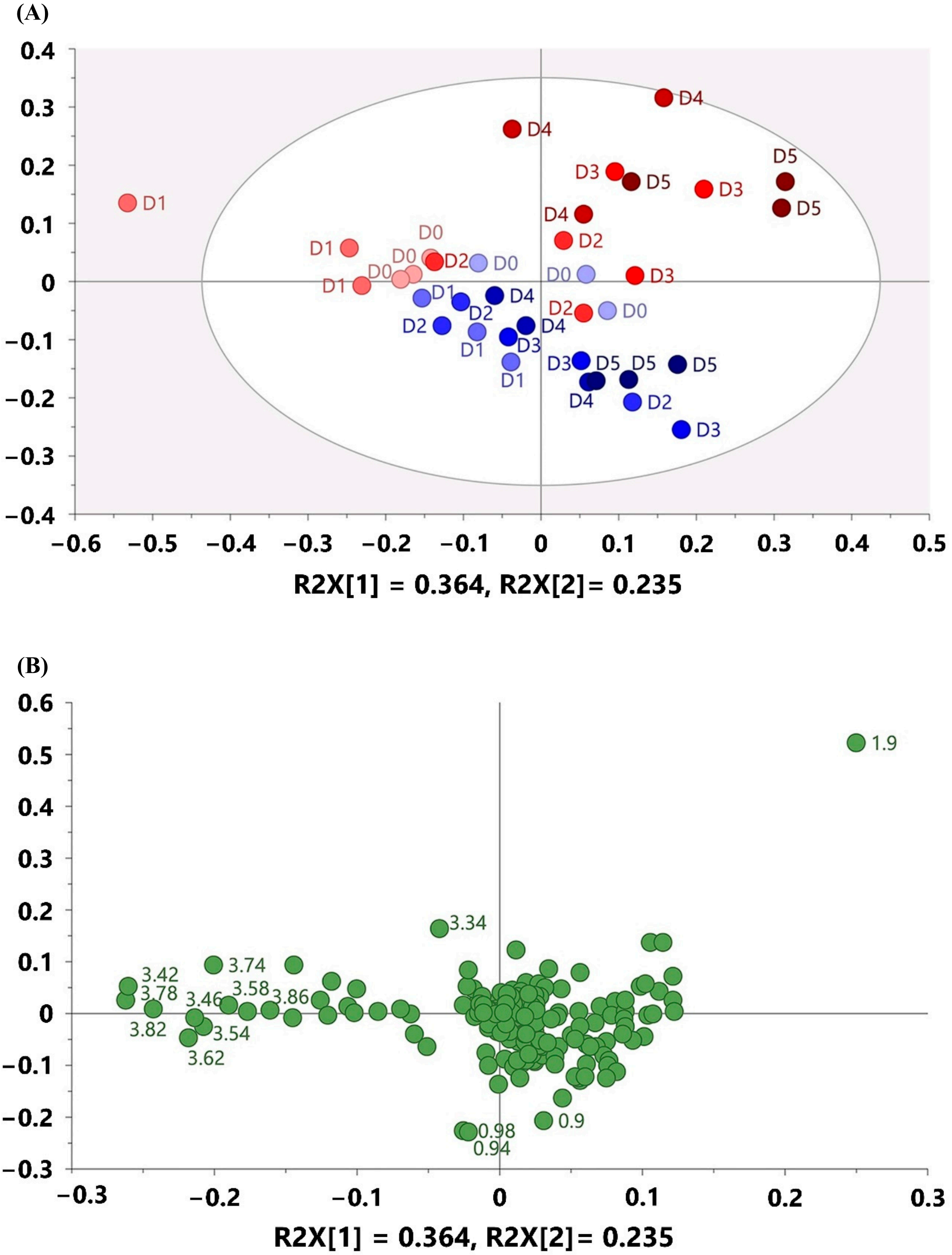
(A)
D0
D1
D2
D3
D4
D5
R2X[1] = 0.364, R2X[2]= 0.235
(B)
1.9
3.34
3.42
3.74
3.78
3.58
3.46
3.86
3.82
3.54
3.62
0.98
0.94
0.9
R2X[1] = 0.364, R2X[2]= 0.235

(C)

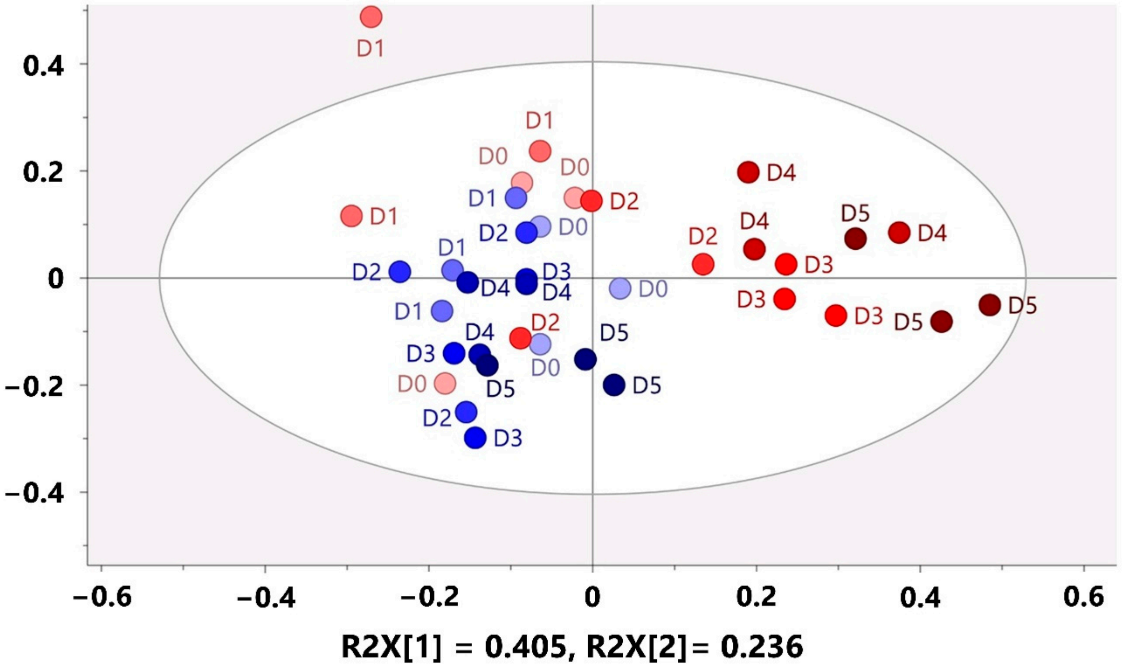

(D)

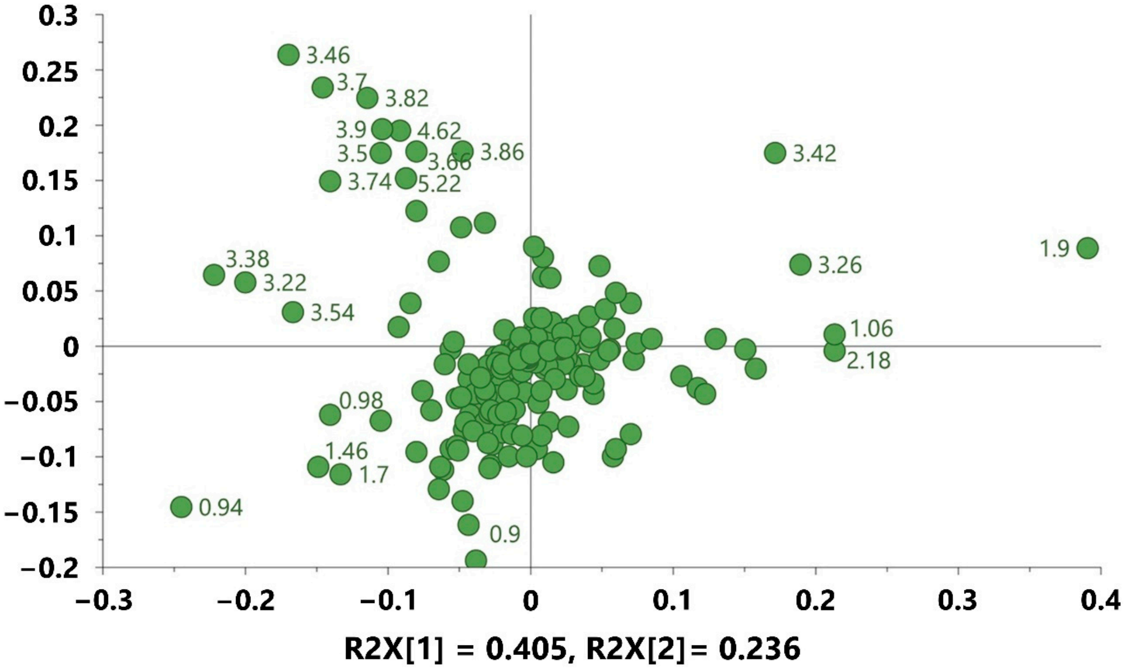

Figure S3. (A) PCA score plot of mice feces of control group (blue) and DSS group (red) from day 0 to day 5 acquired on 60 MHz NMR spectrometer, PC1=36.4%, PC2=23.5%; (B) loading plot of figure S2A; (C) PCA score plot of mice feces of control group (blue) and DSS group (red) from day 0 to day 5 acquired on 800 MHz NMR spectrometer, PC1=40.5%, PC2=23.6%; (D) loading plot of figure S2C.

The depth of the color in the score plots increased as the cultivation time progressed. The R2X[1] and R2X[2] represent the first principal component the second principal component, respectively.

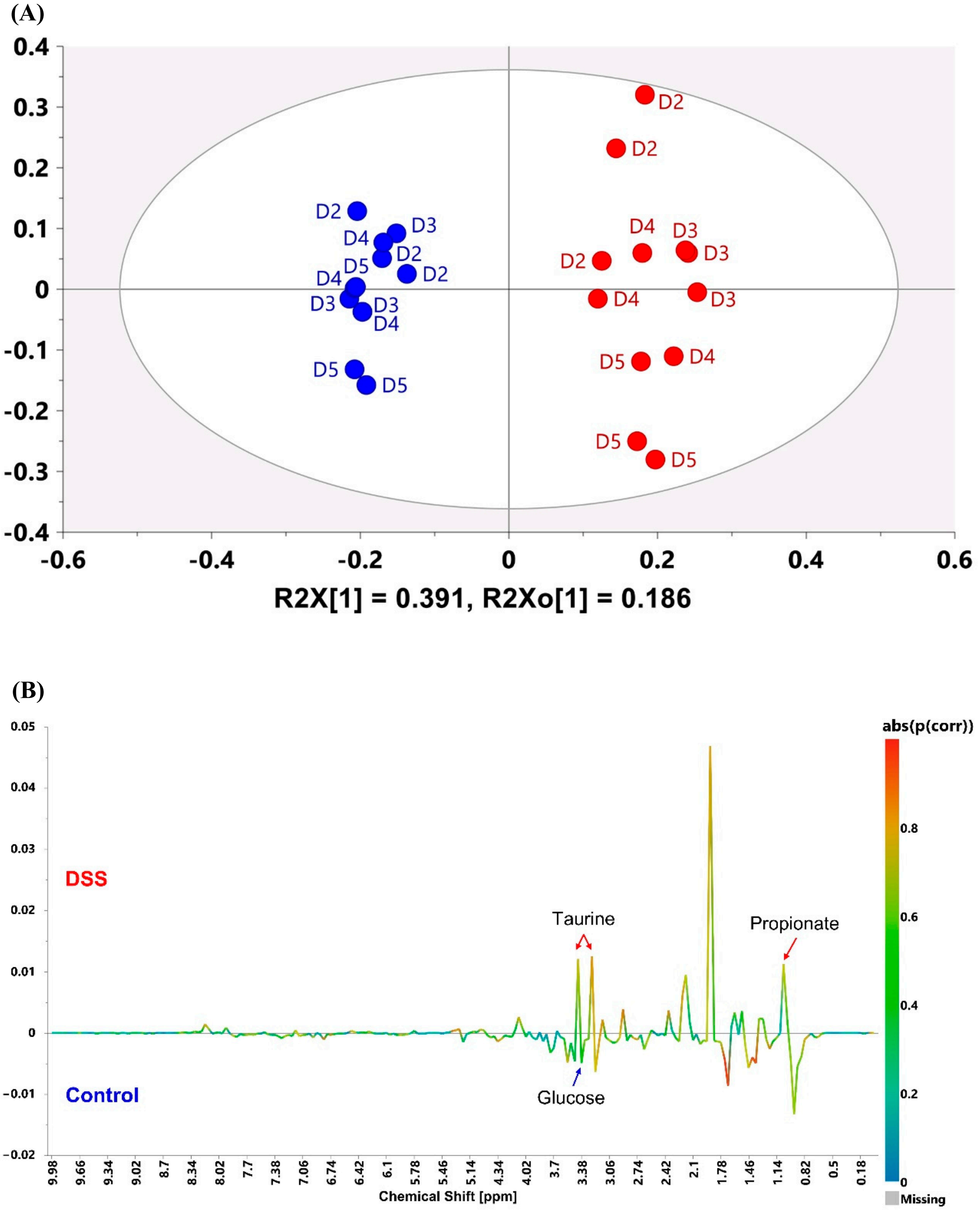

Figure S4. (A) OPLS-DA score plot of mice feces of control group (blue) and DSS group (red) from day 2 to day 5 acquired on 800 MHz NMR spectrometer; (B) OPLS coefficient plot (S-line) of Fig. S3A. The top end with positive value illustrates the increased relative intensity of bins with DSS-treatment while negative represent the decreased relative intensity in the DSS group. The color is associated with the significance of variables in separating the groups as shown on the right side of the plot, where the absolute value of the correlation coefficients was shown.

(A)

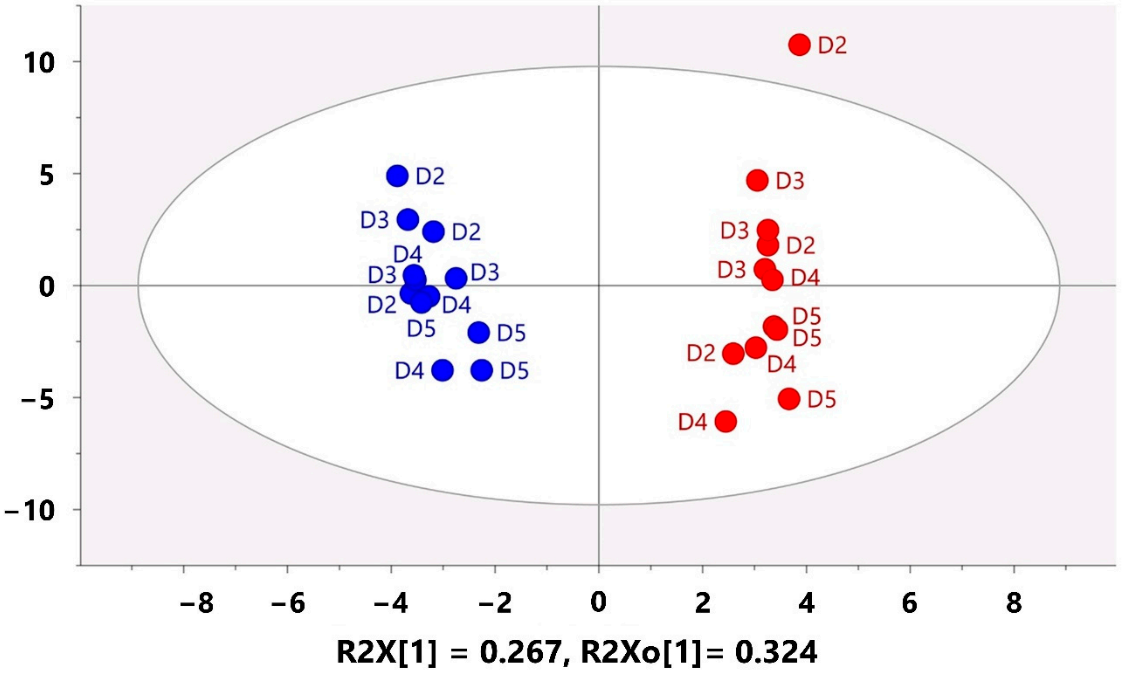

(B)

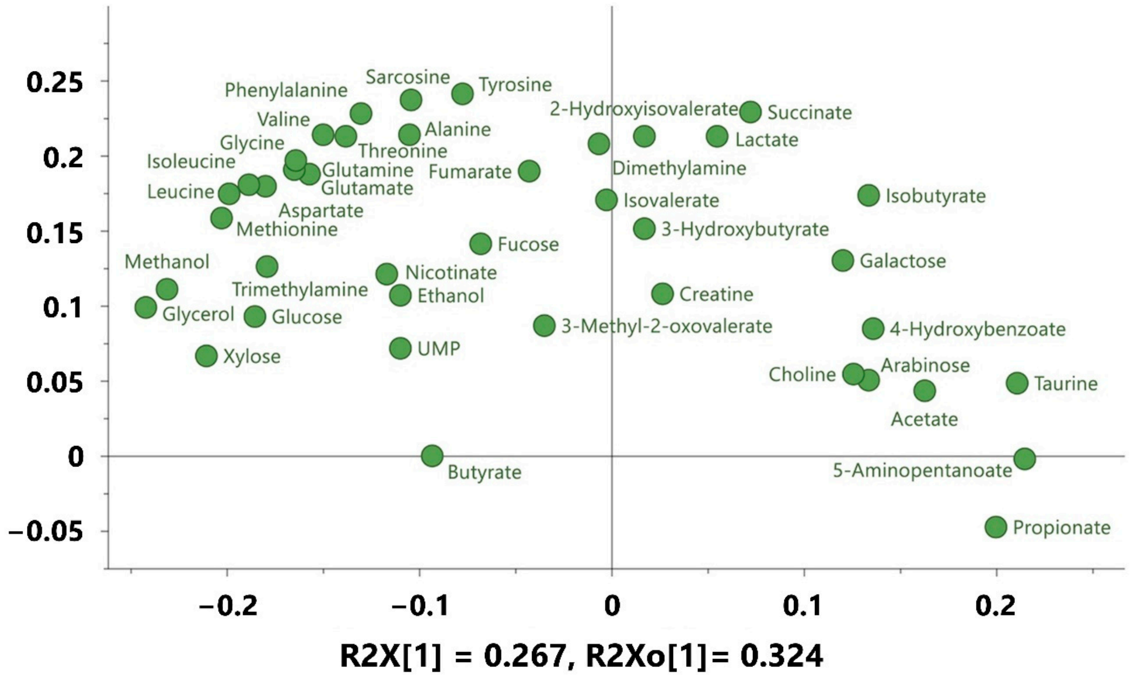

Figure S5. (A) OPLS-DA score plot of concentration of metabolites quantified by Chenomx Profiler in mice feces of control group (blue) and DSS group (red) from day 2 to day 5 acquired on 800 MHz NMR spectrometer; (B) loading plot of figure S4A.

(A)

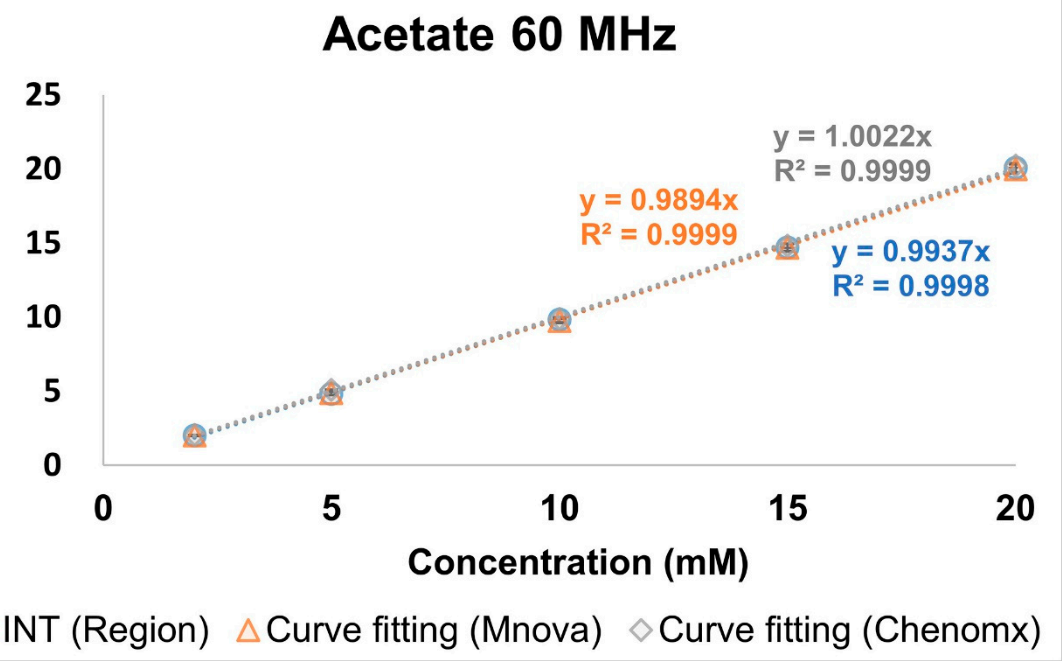

(B)

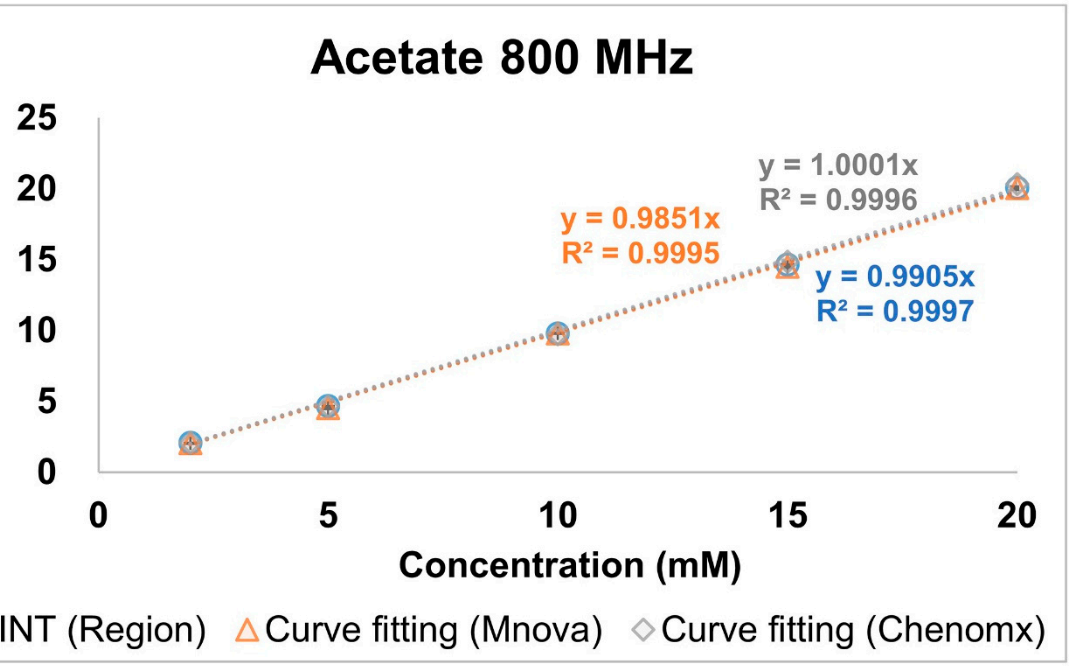

Figure S6. Calibration curve of acetate standard sample measured by 60 MHz (A) and 800 MHz (B) spectrometer. The concentration of acetate was quantified by three methods.

INT (Region): TSP-normalized integration method by manual selection of chemical shift region; Curve fitting (Mnova): the "Generalized Lorentzian" (GL) peak shape was fitted to the spectral line and modified, followed by TSP-normalized integration for the GL peak using the Mnova; Curve fitting (Chenomx): the peak shape was pre-defined by the signal of TSP, followed by manual fitting by referring to the in-house prepared 60 MHz database or the Chenomx built-in 800 MHz database.

Table S1. Comparison of three quantification methods for the concentration of acetate (mM) on 60 MHz NMR spectra.

	**800 MHz**	**60 MHz**					
	Curve fitting (Chenomx)	INT (Region)	Error	Curve fitting (Mnova)	Error	Curve fitting (Chenomx)	Error
D0-CT1	14.47	13.69	-5.4%	15.31	5.8%	13.21	-8.7%
D0-CT2	9.99	11.16	11.7%	10.17	1.8%	10.43	4.4%
D0-CT3	14.73	15.86	7.7%	15.77	7.0%	14.46	-1.9%
D0-DSS1	11.12	11.82	6.3%	10.55	-5.1%	10.59	-4.7%
D0-DSS2	7.09	6.82	-3.8%	7.17	1.1%	6.99	-1.5%
D0-DSS3	11.11	11.89	7.0%	10.93	-1.6%	10.05	-9.6%
D1-CT1	7.32	7.61	4.0%	7.43	1.6%	7.13	-2.6%
D1-CT2	9.79	11.55	17.9%	10.26	4.8%	9.62	-1.7%
D1-CT3	9.40	9.46	0.7%	9.17	-2.4%	8.79	-6.5%
D1-DSS1	7.10	7.96	12.0%	7.28	2.5%	7.02	-1.1%
D1-DSS2	10.78	11.06	2.7%	10.79	0.2%	9.67	-10.3%
D1-DSS3	8.94	8.86	-0.8%	8.85	-0.9%	7.91	-11.5%
D2-CT1	7.47	8.05	7.7%	7.47	0.0%	7.24	-3.1%
D2-CT2	10.17	11.56	13.7%	10.28	1.0%	9.90	-2.7%
D2-CT3	9.99	10.94	9.5%	9.58	-4.1%	9.23	-7.6%
D2-DSS1	10.52	11.93	13.4%	10.29	-2.1%	11.20	6.4%
D2-DSS2	9.29	10.02	7.9%	9.93	6.9%	9.77	5.2%
D2-DSS3	9.33	10.39	11.4%	9.34	0.2%	9.21	-1.2%
D3-CT1	8.13	9.00	10.7%	8.23	1.3%	7.56	-7.0%
D3-CT2	9.09	10.60	16.6%	8.46	-6.9%	8.65	-4.9%
D3-CT3	8.83	9.81	11.1%	8.90	0.8%	8.48	-4.0%
D3-DSS1	8.96	9.71	8.4%	8.96	0.1%	9.39	4.8%
D3-DSS2	12.55	13.40	6.8%	11.90	-5.1%	13.57	8.1%
D3-DSS3	12.08	13.43	11.2%	11.54	-4.5%	11.92	-1.3%
D4-CT1	7.15	7.31	2.2%	7.52	5.2%	7.32	2.5%
D4-CT2	7.69	8.42	9.5%	7.97	3.7%	7.90	2.7%
D4-CT3	7.49	7.68	2.6%	7.34	-2.0%	7.82	4.4%
D4-DSS1	7.47	7.91	5.9%	7.88	5.5%	8.07	8.1%
D4-DSS2	13.86	14.60	5.3%	13.51	-2.6%	14.04	1.3%
D4-DSS3	12.29	12.42	1.1%	12.14	-1.2%	12.48	1.6%
D5-CT1	6.07	6.20	2.2%	6.23	2.7%	5.70	-6.2%
D5-CT2	10.00	9.10	-9.0%	9.49	-5.1%	8.92	-10.8%
D5-CT3	9.51	9.90	4.1%	9.51	0.0%	8.61	-9.5%
D5-DSS1	13.12	13.72	4.6%	12.95	-1.3%	13.38	2.0%
D5-DSS2	13.43	15.17	13.0%	14.45	7.6%	12.84	-4.4%

D5-DSS3	10.69	10.42	-2.6%	11.13	4.1%	10.55	-1.3%

The concentration of acetate quantified by curve fitting method using Chenomx Profiler based on 800 MHz data was used as reference. Then the accuracy of each method for quantifying the concentration of acetate based on 60 MHz NMR spectra was assessed by the percentage error, which was calculated by dividing the reference by the difference between quantified result (60 MHz) and the reference.

INT (Region): TSP-normalized integration method by manual selection of chemical shift region; Curve fitting (Mnova): the "Generalized Lorentzian" (GL) peak shape was fitted to the spectral line and modified, followed by TSP-normalized integration for the GL peak using the Mnova; Curve fitting (Chenomx): the peak shape was pre-defined by the signal of TSP, followed by manual fitting by referring to the in-house prepared 60 MHz database or the Chenomx built-in 800 MHz database.

Table S2. Comparison of integration methods for quantifying the concentration of acetate (mM) on 800 MHz NMR spectra.

	800 MHz				
	Curve fitting (Chenomx)	INT (Region)	Error	Curve fitting (Mnova)	Error
D0-CT1	14.47	14.80	2.2%	14.37	-0.7%
D0-CT2	9.99	10.92	9.3%	10.67	6.8%
D0-CT3	14.73	15.28	3.7%	14.19	-3.7%
D0-DSS1	11.12	10.95	-1.5%	11.88	6.8%
D0-DSS2	7.09	7.63	7.6%	7.39	4.1%
D0-DSS3	11.11	12.05	8.5%	11.25	1.2%
D1-CT1	7.32	7.89	7.9%	7.84	7.2%
D1-CT2	9.79	10.56	7.8%	10.48	7.0%
D1-CT3	9.40	9.36	-0.4%	9.91	5.5%
D1-DSS1	7.10	7.28	2.6%	7.19	1.2%
D1-DSS2	10.78	11.21	4.0%	11.05	2.6%
D1-DSS3	8.94	8.84	-1.0%	8.51	-4.7%
D2-CT1	7.47	7.68	2.8%	7.32	-2.0%
D2-CT2	10.17	10.72	5.4%	10.23	0.6%
D2-CT3	9.99	9.95	-0.4%	9.47	-5.2%
D2-DSS1	10.52	10.54	0.2%	11.04	4.9%
D2-DSS2	9.29	9.75	5.0%	9.55	2.8%
D2-DSS3	9.33	9.78	4.9%	9.61	3.0%
D3-CT1	8.13	8.19	0.8%	8.45	4.0%
D3-CT2	9.09	8.86	-2.5%	9.20	1.2%
D3-CT3	8.83	8.87	0.5%	8.73	-1.2%
D3-DSS1	8.96	9.31	4.0%	9.23	3.0%
D3-DSS2	12.55	12.87	2.6%	13.49	7.5%
D3-DSS3	12.08	12.60	4.3%	13.03	7.9%
D4-CT1	7.15	7.34	2.8%	7.13	-0.3%
D4-CT2	7.69	8.15	6.0%	8.13	5.8%
D4-CT3	7.49	8.03	7.2%	7.93	5.9%
D4-DSS1	7.47	7.28	-2.5%	7.87	5.4%
D4-DSS2	13.86	14.89	7.4%	14.69	6.0%
D4-DSS3	12.29	12.49	1.7%	13.14	6.9%
D5-CT1	6.07	6.07	0.0%	6.01	-0.9%
D5-CT2	10.00	10.06	0.6%	9.85	-1.5%
D5-CT3	9.51	9.57	0.6%	9.36	-1.5%
D5-DSS1	13.12	12.96	-1.2%	13.95	6.3%
D5-DSS2	13.43	14.01	4.3%	12.50	-6.9%
D5-DSS3	10.69	11.51	7.7%	11.62	8.7%

The concentration of acetate quantified by curve fitting using Chenomx Profiler based on 800 MHz data was used as reference. Then the accuracy of two integration methods for quantifying the concentration of acetate based on 800 MHz NMR spectra was assessed by the percentage error.
INT (Region): TSP-normalized integration method by manual selection of chemical shift region; Curve fitting (Mnova): the GL peak shape was fitted to the spectral line and modified, followed by TSP-normalized integration for the GL peak using the Mnova; Curve fitting (Chenomx): the peak shape was pre-defined by the signal of TSP, followed by manual fitting by referring to the Chenomx built-in 800 MHz database.

Table S3. Paired differences of each method for quantifying the concentration of acetate (mM) in the mice fecal sample based on 60 MHz NMR spectra with the routine method (800 MHz curve fitting using Chenomx).

**Quantification methods**	**Routine method**	**Paired differences**					**MAE**
		mean	std. dev.	95% CI		t	
				lower	upper		
60 MHz INT (Region)	800 MHz Curve fitting (Chenomx)	0.623	0.636	0.408	0.839	2.03	0.751
60 MHz Curve fitting (Mnova)		0.047	0.424	-0.096	0.191		0.316
60 MHz Curve fitting (Chenomx)		-0.206	0.560	-0.395	-0.016		0.484

INT (Region): TSP-normalized integration method by manual selection of chemical shift region; Curve fitting (Mnova): the “Generalized Lorentzian” (GL) peak shape was fitted to the spectral line and modified, followed by TSP-normalized integration for the GL peak using the Mnova; Curve fitting (Chenomx): the peak shape was pre-defined by the signal of TSP, followed by manual fitting by referring to the in-house prepared 60 MHz database or the original Chenomx 800 MHz database. The concentration of acetate quantified by curve fitting method using Chenomx Profiler based on 800 MHz data was used as reference. CI: confidence interval; MAE: mean absolute error.

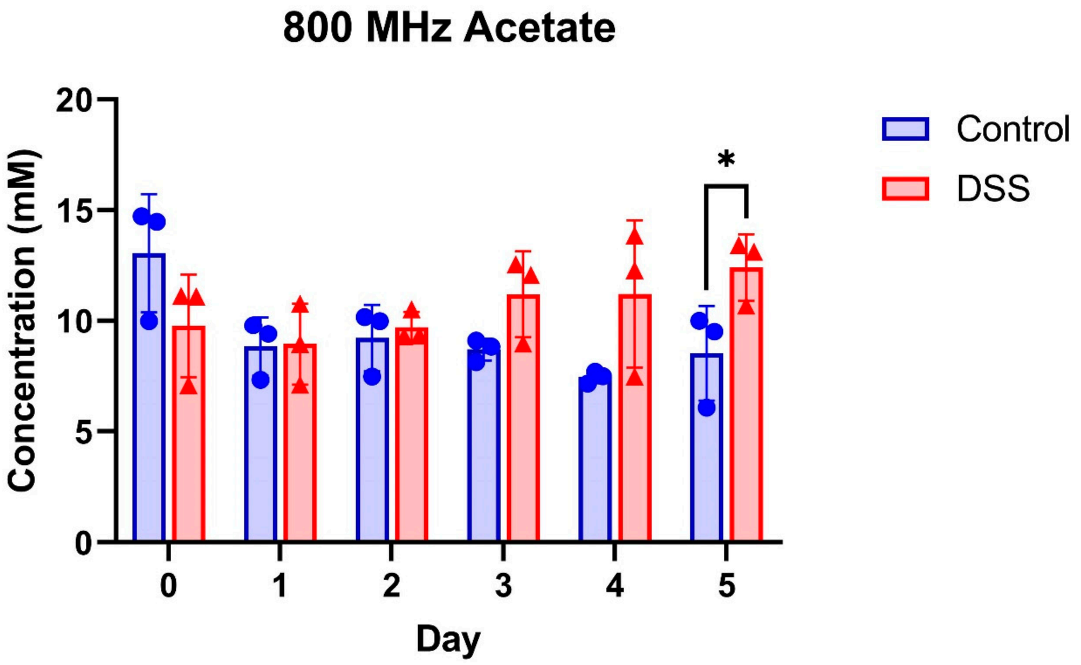

Figure S7. The concentration of acetate in mouse fecal samples quantified by 800 MHz spectra using the "Curve fitting (Chenomx)" method. Welch's unequal variances t-test was used for the comparison. *: $p<0.05$.
